# Spatiotemporal patterns in air pollution and sound in Dhaka, Bangladesh

**DOI:** 10.1038/s41598-025-12815-9

**Published:** 2025-09-25

**Authors:** Martha Lee, Anisur Rahman Bayazid, Lauren Rosenthal, Riaz Hossain Khan, Raphael Arku, Benjamin Barratt, Zahidul Quayyum, Jill Baumgartner

**Affiliations:** 1 Department of Equity, Ethics, and Policy, McGill University, Montreal, Canada; 2 Environmental Research Group, MRC Centre for Environment and Health, Imperial College London, London, UK; 3 BRAC James P Grant School of Public Health, BRAC University, Dhaka, Bangladesh; 4 Department of Epidemiology, Biostatistics, and Occupational Health, McGill University, Montreal, Canada; 5 Department of Environmental Health Sciences, School of Public Health and Health Sciences, University of Massachusetts, Amherst, USA; 6 McGill Centre for Climate Change and Health, McGill University, Montreal, Canada

**Keywords:** Black carbon, Fine particulate matter, Inequalities, Intermittency ratio, PM_2.5_, Environmental impact, Risk factors

## Abstract

High air pollution and sound in Dhaka pose major health risks, yet limited high-resolution data hinders epidemiologic assessments and interventions. We measured fine particulate matter (PM₂.₅), black carbon (BC), and sound (dBA) at 70 locations across Dhaka during the 2023 dry (Jan–Mar) and wet (Jul–Sep) seasons, with continuous monitoring at 8 fixed sites and short-term sampling at 62 rotating sites (3 days/season). Over 1.7 million minutes of PM_2.5_ and 1.2 million minutes of sound were collected, enabling detailed spatial (land use features) and temporal (seasonal, weekly, daily, and diurnal) analyses. Air pollution was four times higher in the dry season, with commercial/industrial areas and transportation corridors having the highest levels, particularly at night. Sound levels varied less temporally, and were highest in transportation corridors, mixed-use areas, and commercial hubs. Air pollution and sound across the city exceeded international guidelines. These findings provide critical evidence to inform targeted policy and interventions.

**Supplementary Information:**

The online version contains supplementary material available at 10.1038/s41598-025-12815-9.

## Introduction

Cities drive economic growth and innovation, but the influx of people, traffic, and industry can place enormous pressure on local environments^1,2^. Dhaka, Bangladesh (pop. 24.7 million)^3^, is one of the world’s most densely populated and fastest-growing megacity, with some of the highest recorded levels of air pollution and noise^4^. Ambient fine particulate matter (PM_2.5_) in Dhaka is 12–18 times higher than the World Health Organization’s (WHO) guideline (5 µg/m^3^)^5^, with annual averages ranging from 63.6 to 91.9 µg/m^3^ between 2010 and 2019^6^. The few studies of environmental noise report levels from 57 to 110 dBA^7–9^, often exceeding the highest permissible area-based noise guidelines for Bangladesh (75 dBA in daytime and 70 dBA in nighttime in industrial areas)^10^ and the WHO European Region (54 dBA for railway noise)^11^.

Health consequences of air pollution and noise are well-established. Exposure to air pollution increases risk of early mortality^12^ and the development of cardio-respiratory diseases^13^, several types of cancer^14^, and neurological disorders^15^. Epidemiologic studies in North America and Europe associated transportation noise with sleep disturbance, annoyance, impaired neuro-cognitive functioning, and cardiovascular diseases^16^. Dhaka’s high levels of air pollution and noise likely pose large population health risks. However, limited high-resolution data prevent epidemiologic studies and health risk assessments, and poses a barrier for identifying and monitoring local emissions hotspots and for designing and evaluating mitigation strategies^17^. All of which were effectively used to inform air quality mitigation strategies in North America, Europe, and other parts of Asia. Thus, there is a significant unmet need to undertake rigorous scientific research on community air quality and noise to support public health policy and regulatory decisions.

We conducted a comprehensive field campaign, measuring seasonal air pollution and sound across 70 diverse locations that captured the city’s diversity of land use and environmental conditions. To our knowledge, this represents the most spatially-resolved empirical study of air pollution and sounds levels in Dhaka to date. Our findings aim to inform ongoing and future environmental assessments, city planning initiatives, and policy solutions tailored to Dhaka’s specific context and pollution sources.

### Study setting

Dhaka is the political capital and economic hub of Bangladesh and is experiencing rapid, often unplanned, growth to absorb its estimated 1500 to 2000 daily migrants^18^. The Dhaka metropolitan area covers ~ 306 km^2^ and is among the world’s most densely populated urban areas, with an estimated population density of 27,599 persons/km^2^ in 2015^19^ and an anticipated population increase to 35 million by 2050^18^. The city’s tropical monsoon climate is characterized by seasonal increases in rainfall that impact air pollution levels and sources. The months of November to March are generally referred to as the “dry season”, as rainfall is at its lowest, where air quality is impacted by brick kiln operations and regional air pollution in addition to local sources like traffic, construction, and industry that also contribute to environmental sound. The months of May to September are considered the “wet season”, where regional and brick kiln pollution decrease but local sources persist^4,20^.

## Results

We captured 1,720,945 min of real-time PM_2.5_ data during 1,193 24-h measurement days across 297 sensor deployments (fixed = 173; rotating = 124) over 124 unique sampling days. Thirty-four sensor deployments (11%) did not achieve within 10 h of target duration (fixed = 33; rotating = 1), mostly due to battery failure or, in one instance, monitor theft. These issues resulted in 76 missed 24-h measurement-days. Gravimetric (filter-based) PM_2.5_ and black carbon (BC) measurements were obtained for 1,170 24-h measurement-days (97% completion), with 62 missing due to battery failure, filter overloading, or monitor theft. There were no instances of simultaneous sensor and gravimetric monitors failure.

We also recorded 1,192,527 min of sound data during 814 24-h measurement-days over 121 unique sampling days. Of the 193 attempted deployments, 179 (93%) achieved within 10 h of the sampling targets. Missing data (*n* = 46 24-h measurement days) resulted from battery or sensor failure. Two rotating site deployments failed completely and were excluded from the analysis.

### Temporal patterns exposure

Average PM_2.5_ and BC concentrations were approximately four times higher in the dry season than the wet season (PM_2.5_: 195 versus 54 µg/m^3^; BC: 13.5 versus 2.9 µg/m^3^), though BC/PM_2.5_ ratios were relatively consistent (7.0% and 5.8% in dry and wet seasons, respectively) (Table 1). All sites exceeded the WHO health-motivated 24-h PM_2.5_ guideline of 15 µg/m^5^. PM_2.5_ levels varied modestly by day of the week. In both seasons, concentrations were generally lower on Thursday (last day of the work week in Bangladesh) and higher on Sundays and Monday (beginning of the work week) (Supplementary Fig. 1; Supplementary Table 2).

**Table 1:** Annual and seasonal levels of temporally-adjusted^a^ PM_2.5_ and black carbon (BC) in Dhaka, by site type and land use category. Data are expressed as the group mean of site averages for each category with standard deviation in parentheses^b^. Annual values are the average of the seasonal means at each site.

Site type (no. of sites)	Season	PM_2.5_ (µg/m^3^)			BC (µg/m^3^)	BC/PM_2.5_ (%)
		All	Daytime 6:00–20:59	Night-time 21:00–5:59	All	All
All sites (*n* = 70)	Annual Dry Wet	124 (43.3) 195 (45.1) 53.5 (46.6)	116 (37.1) 180 (36.1) 52.2 (42.2)	136 (52.5) 216 (55.9) 55.7 (54.3)	8.21 (2.53) 13.5 (4.32) 2.88 (1.30)	6.38 (1.71) 7.04 (2.25) 5.75 (2.09)
Fixed sites (*n* = 8)	Annual Dry Wet	159 (119) 222 (105) 95.9 (134)	147 (103) 203 (86.5) 90.9 (121)	180 (145) 255 (135) 104 (156)	9.50 (2.34) 15.2 (3.35) 3.82 (1.73)	6.70 (1.73) 7.32 (1.65) 6.09 (2.16)
Fixed sites without Shyampur (*n* = 7)	Annual Dry Wet	117 (7.6) 186 (13.4) 48.6 (4.4)	110 (7.7) 172 (12.2) 48.3 (5.1)	128 (9.4) 208 (18.0) 49.1 (3.6)	8.78 (1.23) 14.3 (2.35) 3.28 (0.88)	7.21 (1.02) 7.72 (1.29) 6.71 (1.38)
Rotating sites (*n* = 62)	Annual Dry Wet	120 (17.7) 191 (30.6) 48.1 (11.7)	112 (13.2) 177 (23.0) 47.3 (11.0)	130 (20.4) 211 (35.0) 49.4 (13.7)	8.03 (2.52) 13.3 (4.41) 2.76 (1.20)	6.33 (1.72) 7.01 (2.33) 5.71 (2.10)
Commercial/industrial (*n* = 16)	Annual Dry Wet	143 (83.8) 210 (73.5) 75.8 (94.9)	133 (72.4) 195 (60.4) 72.0 (85.5)	159 (103) 236 (96.1) 82.2 (111)	9.36 (2.98) 15.2 (5.23) 3.26 (1.57)	6.35 (1.87) 7.41 (2.51) 5.44 (1.97)
Green, blue, open space (*n* = 8)	Annual Dry Wet	113 (7.94) 181 (16.8) 43.9 (5.49)	106 (6.26) 169 (13.0) 43.3 (5.42)	123 (13.5) 202 (29.3) 45.0 (6.17)	7.79 (2.80) 13.4 (5.16) 2.15 (0.56)	6.18 (1.91) 7.42 (2.81) 4.94 (1.28)
Mixed-use zone (*n* = 9)	Annual Dry Wet	116 (12.9) 183 (20.7) 49.3 (14.1)	110 (11.9) 170 (17.4) 49.9 (14.6)	127 (16.4) 206 (29.3) 48.2 (13.8)	8.65 (3.41) 13.7 (5.14) 3.35 (1.86)	7.04 (2.33) 7.58 (3.24) 6.26 (1.96)
Residential (*n* = 19)	Annual Dry Wet	114 (16.4) 185 (31.4) 42.8 (9.21)	108 (14.2) 173 (27.7) 41.9 (8.77)	124 (21.1) 204 (39.8) 44.4 (10.5)	7.16 (1.77) 11.8 (2.97) 2.47 (1.08)	6.18 (1.63) 6.47 (1.42) 5.89 (2.70)
Transportation corridor (*n* = 18)	Annual Dry Wet	128 (21.4) 204 (39.2) 51.5 (6.39)	117 (13.1) 184 (24.9) 50.8 (6.55)	137 (20.2) 222 (36.6) 52.6 (8.10)	8.42 (1.98) 13.8 (3.67) 3.11 (1.06)	6.40 (1.34) 6.90 (2.01) 6.01 (1.87)

^a^Temporal adjustments were made using a weekly-specific temporal adjustment factor (TAF) to correct for the impact of within season temporal trends in ambient PM_2.5_ on our measurements due to variations in deployment dates. ^b^Medians, standard error, and ranges are provided in Supplementary Table 1. BC measurements were missing from four sites annually (Dry: *n* = 2 and Wet: *n* = 2).

**Table 2:** Sound metrics in Dhaka by site type and land use category. Data are expressed as medians (interquartile ranges)^a^. Wet and dry season data were combined for fixed sites.

Land use category (no. of sites)	LA_eq_^b^ (dBA)	L_Day_^c^ (dBA)	L_Night_^d^ (dBA)	IR^e^ (%)	IR_Day_^f^ (%)	IR_Night_^g^ (%)
All sites (*n* = 68)	63.0 (10.3)	64.8 (10.6)	60.0 (12.0)	49.1 (29.9)	47.9 (32.1)	48.4 (28.5)
Fixed sites (*n* = 8)	61.2 (5.1)	62.3 (3.8)	58.6 (10.4)	35.5 (20.7)	36.3 (21.7)	35.1 (20.9)
Rotating sites (*n* = 60)	63.2 (11.0)	64.9 (11.1)	60.2 (12.6)	50.1 (30.2)	48.6 (31.3)	50.9 (25.7)
Commercial/industrial (*n* = 15)	64.5 (6.3)	65.2 (4.4)	60.5 (7.1)	49.2 (25.9)	48.5 (27.2)	52.2 (16.4)
Green, blue, open space (*n* = 8)	54.9 (4.4)	56.4 (6.3)	52.4 (3.8)	68.2 (32.6)	67.4 (33.0)	52.8 (34.9)
Mixed-use zone (*n* = 9)	72.9 (3.9)	73.7 (3.9)	68.9 (7.9)	35.5 (31.3)	32.8 (33.8)	36.7 (14.9)
Residential (*n* = 19)	60.8 (3.7)	61.2 (3.6)	55.9 (2.4)	61.3 (16.8)	55.8 (15.4)	64.2 (23.2)
Transportation corridor (*n* = 17)	69.8 (9.2)	71.7 (9.1)	66.9 (7.2)	35.3 (17.2)	32.2 (17.9)	41.5 (10.8)

^a^Means, standard error, and ranges are provided in Supplementary Table 3; ^b^LA_eq_ is the median equivalent continuous sound level. ^c^L_day_ is the median equivalent continuous daytime sound levels between 6:00 and 20:59. ^d^L_night_ is the median equivalent continuous nighttime sound levels between 21:00 and 5:59. ^e^IR is the median intermittency ratio. ^f^IR_Day_ is the median daytime intermittency ratio between 6:00 and 20:59. ^g^IR_Night_ is the median nighttime intermittency ratio between 21:00 and 5:59.

The median sound level (LA_eq_) across sites was 63.0 dBA (IQR: 10.3; range: 50.5, 84.0), exceeding Dhaka’s residential noise guideline (55 dBA) but below its industrial zone guideline (75 dBA) for daytime (Table 2; Supplementary Table 4)^10^. Only 1.9% of daytime and 2.7% of nighttime measurement minutes were below the WHO European guidelines for traffic noise (53 dBA daytime; 45 dBA nighttime)^11^. Based on fixed site data, sound levels were similar across seasons and there was less than 1 dBA variation in average sound across days of the week (Supplementary Fig. 2; Supplementary Table 5).

**Fig. 1:**
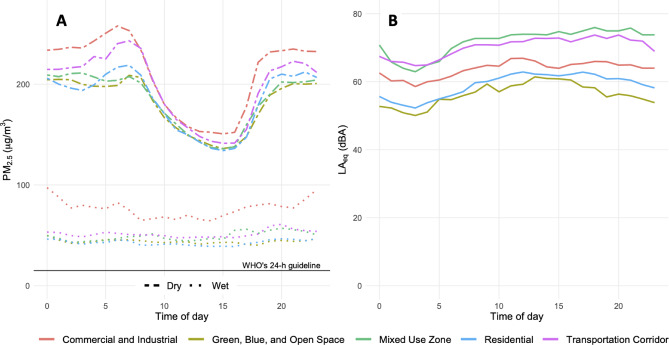
Diurnal patterns of **(A)** average air pollution by season and **(B)** sound in Dhaka. Air pollution data show the temporally-adjusted hourly mean PM_2.5_ (µg/m^3^) by season and land use category. Sound data show the median LA_eq_ for each hour of the day by land use category. Fifteen-minute rolling average values are provided in Supplementary Fig. 3.

 Diurnal patterns for air pollution and sound were distinct and generally consistent across land use categories (Fig. 1). Average PM_2.5_ levels were higher at night than during the day in the dry season (216 versus 180 µg/m^3^), while in the wet season, day and nighttime levels were similar (56 versus 52 µg/m^3^). However, at commercial and industrial sites, nighttime PM_2.5_ remained elevated in the wet season (Table 1), largely driven by the very high nighttime PM_2.5_ levels at the industrial (fixed) site of Shyampur (wet season nighttime mean of 491 µg/m^3^ versus daytime mean of 389 µg/m^3^). Sound followed an inverse pattern, with higher median levels during the day (65 dBA; IQR: 10.6) than at night (60 dBA; IQR: 12.0) (Table 2). We observed distinct peaks in sound levels that corresponded with calls to prayer (Supplementary Fig. 3).

The intermittency ratio (IR), a measure of the disruptiveness of sound events compared with background levels, showed little difference on average between day and night (medians of 47.9 verses 48.4% for day and nighttime respectively) (Table 2). However, site-specific variation was substantial: at 13 sites, daytime IR exceeding nighttime IR by > 5% (range: 5.5 to 34.6%), while at 36 sites, nighttime IR was > 5% higher (range: 6.6 to 38.5%). These findings suggest that the diurnal IR patterns depend on site-specific characteristics. Generally, transportation and residential sites had higher nighttime IRs, while green, blue, and open (GBO) spaces had higher daytime IRs. These patterns were consistent across different IR threshold in sensitivity analyses (Supplementary Table 6; Supplementary Note 7).

### Spatial patterns

**Fig. 2:**
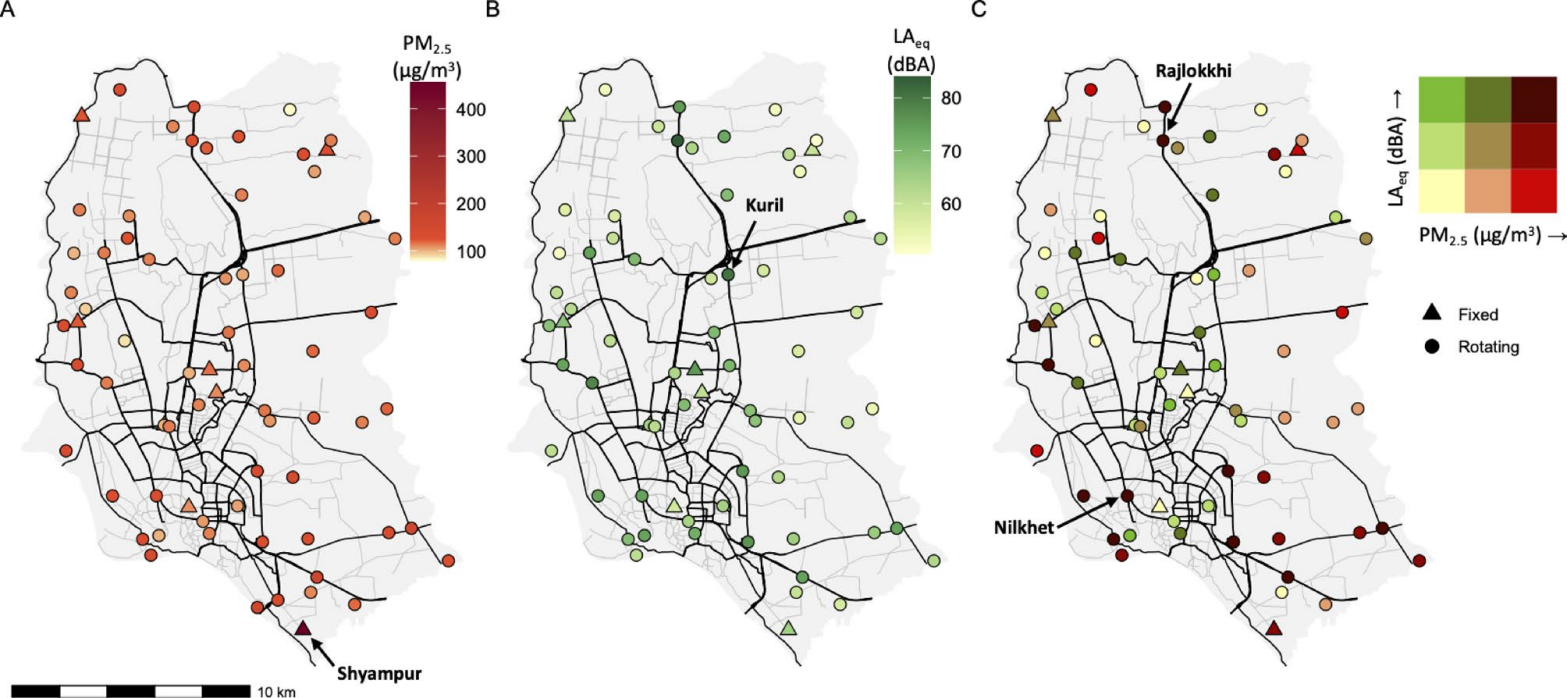
Spatial variation in average air pollution **(A)**, sound **(B)**, and their combination **(C)** in Dhaka. The mean PM_2.5_ concentrations are the mean average of temporally-adjusted site means from both seasons. The LA_eq_ (B) represents the site median. The bivariate map (C) assigns each site into one of nine combined pollution categories based on PM_2.5_ and sound tertiles. Identified hotspots for PM_2.5_ and sound are also labelled. Tertiles were calculated from quantiles with breaks at 33.33% and 66.66%.

 Sound levels were highest in the urban core, whereas PM_2.5_ concentrations peaked in peripheral industrial zones, especially in southern Dhaka (Fig. 2). Estimated annual PM_2.5_ ranged from 81 to 453 µg/m^3^ and BC from 2.9 to 14.5 µg/m^3^. The Shyampur fixed site, located in an informal settlement near an industrial area, recorded the highest PM_2.5_ (range of daily means: 73 to 1128 µg/m^3^). BC levels were also high in Shyampur (dry/wet season averages of 21.5/7.6 µg/m^3^), though less extreme than PM_2.5_.

**Fig. 3:**
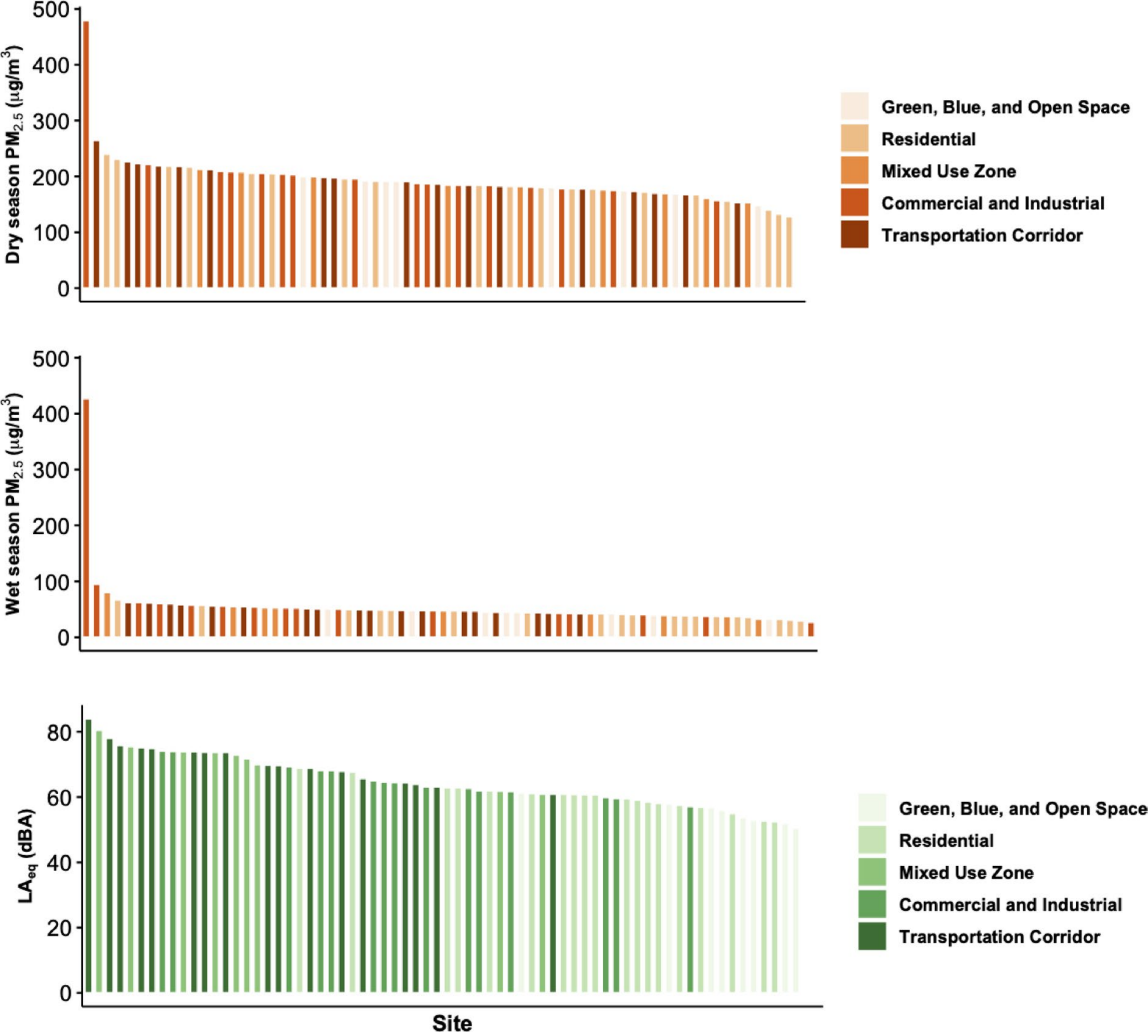
Descending bar graph of site- and season-specific mean temporally-adjusted PM_2.5_ (µg/m^3^) and median LA_eq_ (dBA) by land use category in Dhaka.

 Air pollution and sound were higher in transportation, commercial, and industrial zones, and lower in residential and GBO sites (Table 1; Fig. 3). We observed more notable differences in PM_2.5_ by land use type in the wet season (p-value = 0.007) than dry season (p-value = 0.29). Median LA_eq_ ranged from 51 to 84 dBA across sites (median: 63) (Table 2; Supplementary Table 3). Mixed-use sites had the highest median LA_eq_ (73 dBA), followed by transportation corridors (70 dBA), commercial and industrial sites (65 dBA), residential areas (61 dBA), and GBOs (55 dBA) (p-value ≤ 0.001) (Table 2). According to Bangladesh’s land use-specific Noise Pollution Control Rules, 84% of residential sites exceeded daytime limits, and all residential sites exceeded nighttime limits. In commercial/industrial areas, 7% exceeded daytime and 27% exceeded nighttime limits, while all mixed-use sites exceeded both day and nighttime limits by 1–59% (Supplementary Table 4)^10^.

IR values also varied by land use (p-value = 0.002). Residential and GBO sites, which had the lowest average sound levels, exhibited the highest IRs (medians: 61.3% and 68.2%, respectively) compared with other land use categories (range of medians: 35.3–49.2%) (Table 2). The IR values were lower at fixed sites than at rotating sites, despite similar average sound levels. This discrepancy may reflect the underrepresentation of fixed monitors in higher IR land use types, such as residential (*n* = 1) and GBO zones (*n* = 2).

Site-averaged PM_2.5_ and sound levels were weak-to-moderately correlated (Spearman *r*_dry_=0.35; *r*_wet_=0.54) (Supplementary Fig. 4). Identified co-hotspots included the Shyampur informal settlement (PM_2.5_: 453 µg/m^3^; sound: 65 dBA), the mixed-use sites at Kuril (PM_2.5_: 103 µg/m^3^; sound: 81 dBA) and Nilkhet (PM_2.5_: 131 µg/m^3^; sound: 74 dBA), and a transportation corridor in Rajlokkhi (PM_2.5_: 124 µg/m^3^; sound: 84 dBA) (Fig. 2).

## Discussion

Dhaka, a rapidly growing megacity of over 24 million people, experiences high levels of air pollution and noise that pose serious public health risks. Yet, there is limited city-wide evidence on the levels, patterns, and sources to support effective management strategies^7,21^. At most monitoring sites in this city-wide study, both air pollution and nighttime sound levels exceeded national and international guidelines. Air pollution was markedly higher during the nighttime, in the dry season, and in commercial and industrial areas. In contrast, sound levels exhibited less temporal variability but were highest in commercial markets and transportation corridors. We did not observe a strong overall correlation between sound and air pollution, highlighting the need for tailored mitigation strategies, though traffic may offer a shared point of intervention.

Air pollution peaked during the dry season, consistent with prior studies in Dhaka, and largely attributed to transboundary pollution from Nepal and India, unfavorable meteorological conditions (lower wind speeds, reduced mixing layer height, and minimal rainfall), and local sources such as brick kilns and biomass burning^4,20,22–24^. In contrast, sound remained consistently high year-round, with sources being exclusively local, including traffic, construction, commercial activities, and daily calls to prayer.

We observed minimal variation in air pollution or sound across different days of the week. This aligns with a prior study in Dhaka suggesting that weekend delivery truck traffic offsets reductions in commuter traffic^20^. Such patterns resemble those observed in other rapidly urbanizing cities like Accra^25,26^, but differ from cities in North American and European, where levels of sound^27,28^ and air pollution^29,30^ are generally higher on weekdays due to structured workweek commuting.

Nighttime PM_2.5_ levels were consistently elevated, a finding consistent with previous studies in Dhaka^20,24^ and other cities^31^. This is likely due to the formation of a shallow and stable nocturnal boundary layer that limits vertical dispersion and traps pollutants near the surface^31^. This pattern persisted in the wet season but was less pronounced due to rainfall and improved dispersion conditions^24^.

Elevated nighttime sound, which can worsen sleep quality and quantity^32^, also showed distinct peaks that aligned with daily calls to prayer. In Dhaka, these are broadcasted through loudspeakers installed on mosque minarets. Similar findings were reported in urban Ghana^26^ and Cameroon^33^, where religious soundscapes also contributed to sound. The median IR at rotating sites in our study (median: 50%, IQR: 30) were similar to those in a similar citywide study in Accra, Ghana (median: 53%, IQR: 16)^26^, though the range of values was larger in our study. Similar trends were observed by site type, with higher IRs at residential sites, likely due to the lower levels of background sound and more sporadic nature of sound sources in these areas. Though our study did not investigate health impacts, previous studies suggest that continuous and intermittent noise may impact health through different mechanisms, with the former activating the sympathetic nervous system and oxidative stress and the latter disrupting sleep and activating stress response^34,35^.

Our observed BC/PM_2.5_ ratios were lower than those reported in Nairobi, Mumbai, and Rio de Janeiro but comparable to levels in Delhi, Seoul, Mexico City, and numerous cities in Europe and North America^36^. The lower ratios in our study might reflect the influence of regional pollution. BC, a carbonaceous pollutant emitted from incomplete combustion and contributor to climate warming^37^, comprised a larger share of PM_2.5_ in the dry season and in commercial and mixed-use areas. Elevated BC/PM_2.5_ ratios at some GBO sites in the dry season likely reflect the impact of nearby brick kilns and biomass burning^38^, while the decline in these ratios in the wet season aligns with reduced activity of these sources.

​​An informal (slum) settlement (Shyampur), located in an industrial area in southern Dhaka, emerged as an air pollution hotspot. This site was located near steel re-rolling mills and other small-scale industry and had high air pollution throughout the study period. Shyampur represents an important focal point for future intervention given its persistently high levels of air pollution and large local population of over 184,000 residents^39^. Mitigation strategies such as stricter emissions controls and the modernization of polluting industries should be considered alongside targeted public health measures for this extremely vulnerable area of Dhaka.

Although no single site was an extreme sound hotspot, several mixed-use areas and transportation corridors consistently recorded high sound levels. Kuril, a major transportation hub with new flyover infrastructure, had high levels of sound but low air pollution relative to other sites in the city. This likely reflects higher vehicle volume combined with improved traffic flow and nearby open space dispersed that helped disperse air pollution. Nilkhet, a dense commercial hub, experienced constant sound levels from heavy pedestrian activity and traffic congestion, including cars, rickshaws, motorcycles, and delivery trucks that navigate its narrow roads and contribute to both sound levels and air pollution. Rajlokkhi, located along the eight-lane Dhaka-Mymensingh highway, recorded the highest sound levels. This major traffic artery sees continuous daytime congestion. Nighttime traffic volume is lower, but high-speed freight trucks and long-haul buses continue to dominate the roadway, sustaining elevated sound levels.

Dhaka’s poor air quality has drawn substantial public attention, prompting several policy and regulatory efforts, including a 2022 ban on two-stroke vehicles and the 2010 Clean Air and Sustainable Environment project which promoted cleaner brick kiln technologies and changes to intersection infrastructure to improve traffic flow^23^. The spatial variation in PM_2.5_ in our study suggests that mitigating local sources could yield measurable benefits to air pollution and can inform policy solutions that targeted Dhaka’s urban context. For example, stricter vehicle emissions standards, expanding low-emission zones, and continued investment in public transit could help mitigate the high levels of pollution measured in transportation, commercial, and industrial sites in our study.

Noise pollution has only recently gained policy attention in Dhaka. Bangladesh established noise limits for different land use types in 2006, though there is limited evidence of their enforcement^40^. In October 2024, the interim government launched a campaign to raise public awareness about noise and phased in a ban on vehicle honking in Dhaka with penalties for the use of horns, aiming to transform the city into a “quiet zone” in 2025^41^. Enforcement remains a challenge^42^, however traffic control does present a feasible entry point for integrated mitigation. Our findings support enforcement and possible expansion of these measures, including speed control, traffic management, and public transit improvements that can reduce both noise and air pollution.

This study has several limitations to consider in future work. First, long-term continuous pollution monitoring at all sites was not possible due to logistic and equipment constraints. Although we applied temporal adjustments to account for variation in pollution concentrations, we may have over- or under-estimated the ‘true’ season-specific averages at rotating sites. We found the mean RMSE across fixed sites by season when comparing three-day temporally-adjusted measurements with the seasonal mean was 22.3 µg/m³ (dry) and 6.4 µg/m³ (wet). Second, diurnal PM_2.5_ trends were measured using light-scattering sensors, which are less accurate at high concentrations^43^. While we found no evidence of non-linearity in sensor-gravimetric comparisons, some measurement error is inevitable. However, any inaccuracies are unlikely to materially affect our conclusions. Third, sound measurements at the rotating sites were limited to three days in the wet season. Although fixed-site data showed no clear seasonal or weekly differences across varied urban sites, we may have missed seasonal patterns at some rotating sites that are relevant to policy. Fourth, battery failures led to some data loss, mainly at fixed sites on the fifth day of measurement. As measurements spanned entire seasons and failures occurred at different days and times throughout, the impact of these missed days on the seasonal mean is likely minimal. Fifth, our source assessments for PM_2.5_ and sound relied on BC components, land use characteristics and expert judgement rather than chemical speciation or sound recordings; an aligned study is addressing these gaps for future publication. Future research could examine the long-term impacts of environmental interventions, investigate how socioeconomic and infrastructural disparities affect exposures, and quantify the health impacts of PM_2.5_ and environmental sound.

Dhaka is a rapidly expanding megacity that is grappling with severe air pollution and noise. This study is one of very few to quantitatively assess the intersection of air pollution and sound and their spatiotemporal distributions, which are understudied in Global South cities^2^. Our measurements indicate that all neighborhoods in Dhaka could benefit from intervention and point to traffic-related policies as a promising entry point for city-wide interventions. We also identified hyper-local hotspots like Shyampur, where industrial emissions drive extreme air pollution, and parts of northern Dhaka where dense traffic and commercial activities contribute to high levels of both air pollution and sound. Our study illustrates an approach for municipalities to prioritize areas for interventions for different health-damaging pollutants and reduce their impacts on this megacity’s large and growing population. It also demonstrates the potential for such environmental data platforms to address questions important in the quest for healthier cities.

## Methods

### Study design

We conducted measurement campaigns from January 18 to March 17 (dry season) and from July 22 to September 24 (wet season) in 2023. In each campaign, air pollution was measured continuously at eight ‘fixed’ sites and for three consecutive days at 62 ‘rotating’ sites. Sound was continuously monitored at fixed sites in the dry season and expanded to all sites in the wet season. This study followed a similar approach to an environmental campaign in Accra, Ghana^44^.

### Site selection and land use classification

**Fig. 4:**
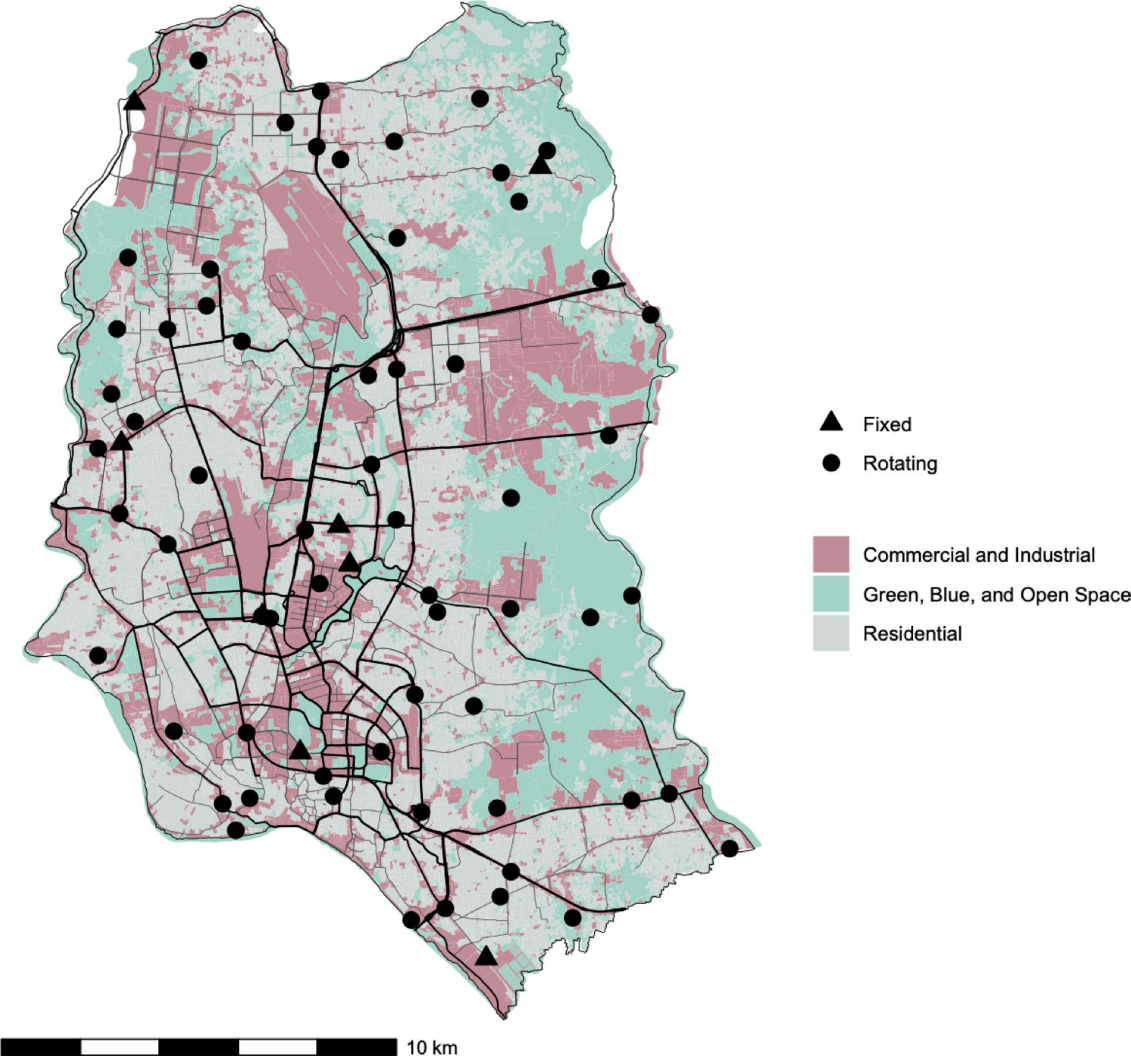
Location of sampling sites, major roads, and land use categories in Dhaka, Bangladesh. Land use categories were adapted from the World Bank land use/land cover dataset (2017) used for initial site selection. The additional category of ‘mixed-use’ used in the analysis was defined for specific sites prior to data collection.

The fixed sites were selected to represent Dhaka’s diverse of environmental features that likely contribute to varying levels of air pollution and sound. The rotating sites were selected via stratified random sampling, where potential locations were randomly distributed throughout Dhaka across strata of land use and population density (Fig. 4).

Each site was assigned a land use classification based on available land use data^45^, visual inspection of satellite and street-view images, and investigator knowledge. The resulting five categories encompass a broad range of anticipated sound and air pollution levels: (1) residential, (2) commercial and industrial, (3) green, blue, and open (GBO) spaces, (4) transportation corridor, and (5) mixed-use. Residential areas included both planned communities and informal settlements. Commercial and industrial areas were a combined category because of the frequent overlap in those activities. Commercial areas had a high density of office complexes, restaurants, and retail stores, whereas industrial zones had more manufacturing, factories, and warehouses. GBO areas were less developed environments primarily characterized by empty plots and/or natural elements such as parks, agricultural land, or waterways. Transportation corridors were areas of high vehicular traffic or train operations. Mixed-use areas were characterized by a combination of activities without a dominant single category (e.g., equal proportions of commercial and residential activities in the same area). The final classification yielded 19 residential sites (1 fixed, 18 rotating), 16 commercial and/or industrial sites (3 fixed, 13 rotating), 8 green, blue, and open spaces (2 fixed, 6 rotating), 18 transportation corridor sites (2 fixed, 16 rotating), and 9 mixed-use rotating sites.

### Real-time and gravimetric measurement of PM_2.5_

We measured PM_2.5_ using co-located gravimetric and sensor-based monitors housed in waterproof enclosure at 3–5 m above ground level. Gravimetric and sensor-based monitors were replaced every 5 days at the fixed sites. Gravimetric measurements were collected using Ultrasonic Personal Aerosol Samplers (UPAS v2, Access Sensor Technologies, USA), attached to an external battery (V50 USB Battery Pack, Voltaic). They sampled air at 1.0 L/min^46^ with a 50% duty cycle, the internal pump would run and then rest in 15 s alternating intervals, to conserve battery power and prevent filter overloading. Monitors housed 37 mm polytetrafluoroethylene (PTFE) filters (2 μm pore size). Airflow was calibrated pre-deployment and measured post-sampling, with all post-sampling flow rates within 5% of the target. Filter samples and blanks (16%, *n* = 49) were stored in sealed Petri dishes and at −18 °C before shipment for gravimetric analysis.

Continuous PM_2.5_ measurements (1 min resolution) were collected using laser-based sensors (Plantower PMS7003, Zefan, Inc., China) with a counting frequency of 98% for particles > 0.5 μm diameter^47^. The sensors were powered by an external battery and were previously deployed in a range of environmental conditions^25,48,49^. To account for known biases in optical, sensor-based measurements, we applied a season-specific correction models using co-located gravimetric reference data over a wide range of representative concentrations (300 µg/m^3^ and 150 µg/m^3^ in dry and wet seasons respectively)^50,51^. We examined potential non-linearity in the optical-gravimetric relationship using correlation plots and fitting statistical splines for each season. As no evidence of non-linearity was observed, we proceeded with season-specific linear regression models (Supplementary Note 1). On average, sensors underestimated gravimetric PM_2.5_ by 36% in the dry season and 2% in the wet season. A separate correction factor was applied to the Shyampur fixed site due to its higher pollutant range. The gravimetric-corrected sensor data were used for analysis.

### Filter analysis for mass and BC

Filters were analyzed for their mass and BC before and after sampling at the Colorado State University Automated Air Analysis Facility (AIRLIFT). Post-sampling, filters were cold chain transported to Colorado and stored in a −20 °C freezer. Prior to mass analysis, filters were conditioned (21–22 °C, 30–34% RH) for at least 24 h, discharged with a polonium source for at least 15 s, and weighed on a microbalance (Mettler Toledo Inc., XS3DU, USA, 1 µg resolution). Filters were weighed in triplicate until the difference between three consecutive weights was < 3 µg^52^. Sampled PM_2.5_ mass was the difference between pre- and post-sampling filter mass. Sampled mass values were blank-corrected using the median value of the field blank filters (2 µg dry season, 4 µg wet season), and concentrations were calculated by dividing the blank-corrected mass by the sampled air volume (Supplementary Note 2).

BC was quantified using an optical transmissometer (SootScan OT21, Magee Scientific, USA) where the difference between in pre- versus post-sampling light attenuation on each filter was converted to mass surface loading using a mass absorption cross section of 16.6 m^2^/g for an 880 nm channel optical BC^53^. We calculated the BC concentrations by subtracting the median field blank values (2.03 dry season, 0.15 wet season), converting the optical measurements to mass, multiplying the surface loadings by the sampled surface area of the filters (8.6 cm^2^), and dividing by the sampled volume of air (Supplementary Note 2).

### Sound level data collection and processing

We measured A-weighted sound levels (dBA) using three sound level meters models with type 1 digital MEMS microphones (NSRT_mk3, NSRT_mk4, and NS110; Convergence Instruments, Montreal, Canada) that were factory-calibrated and co-located before each campaign to ensure their precision and accuracy (Supplementary Note 3). Sound monitors were deployed at the same locations as air pollution devices and programmed to continuously capture sound (dBA) at one-minute intervals for three days at rotating sites (wet season only) or continuously at fixed sites (both seasons). Dry season measurements were limited to fixed sites due to equipment availability. We did not observe any systematic differences in seasonal sound levels at fixed sites (Supplementary Note 4), and thus combined the data from both seasons for statistical analysis. We describe our results as “sound” rather than “noise” to recognize that auditory experiences in Dhaka encompass both desired and undesired or unpleasant sound.

### Statistical analysis

Continuous data from fixed sites showed that air pollution decreased over time in the dry season and was slightly higher in August in the wet season (Supplementary Note 4), which compromises the comparability of the raw rotating site data that were collected at different times. We therefore applied a weekly-specific temporal adjustment factor (TAF) to the gravimetric-corrected PM_2.5_ and BC values^25^, calculated as the ratio of weekly mean PM_2.5_ or BC at fixed sites and the US embassy monitor (www.airnow.gov), for PM_2.5_, to the seasonal mean (excluding Shyampur) (Supplementary Note 5). We calculated season-specific means and standard deviations of temporally-adjusted PM_2.5_ and BC, using the TAF, for each site. These seasonal values were then averaged to estimate an annual means and standard deviations.

The following sound metrics were calculated for each site:

- A-weighted equivalent continuous daily sound levels (LA_eq_ (dBA)).
- A-weighted equivalent continuous daytime sound levels between 6:00 and 20:59 h (L_day_ (dBA)).
- A-weighted equivalent continuous nighttime sound levels between 21:00 and 5:59 h (L_night_ (dBA)).
- Daily intermittency ratio (IR (%)) (calculation in Supplementary Note 6).
- Daytime intermittency ratio between 6:00 and 20:59 h (IR_day_ (%)).
- Nighttime intermittency ratio between 21:00 and 5:59 h (IR_night_ (%)).

The equivalent continuous sound level (LA_eq_) represents the average sound pressure level over time, capturing total sound energy^54^. We calculated overall LA_eq_ and LA_eq_ for daytime and nighttime hours to capture within-day variation. The IR measures the disruptiveness of sound events relative to continuous background levels, which LA_eq_ does not measure. A high IR (%) indicates a high percentage of sound energy is distinct enough from the background levels to qualify as an “event”^55^. For the main analysis, we defined a sound event using a cut-off of + 4 dBA above the site-specific LAeq (overall, daytime, or nighttime), accounting for Dhaka’s high background sound levels. Sensitivity analyses evaluated lower (+ 3 dBA) and higher (+ 5 dBA) cut-off values (Supplementary Table 6; Supplementary Note 7). Summary statistics were based on median values due to the non-linear decibel scale.

We retained all air pollution and sound measurements that captured at least 24 h of measurement. The temporal analysis consisted of seasonal, weekly (for TAF), daily, and diurnal variations in air pollution and sound, and the spatial analysis consisted of summary statistics across sites and by land use type. We estimated the differences in mean air pollution and median sound by season and day of the week using linear regression models with site-specific cluster-robust standard errors^56^. A Spearman correlation matrix was calculated to evaluate the relationship between site averaged air (PM_2.5_, BC, BC/PM_2.5_ ratio) and sound (LA_eq_, IR) measures. Kruskal-Wallis rank sum tests were used to examine the differences by land use types.

All analyses were conducted in R (version 4.4.1).

## Supplementary Information

Below is the link to the electronic supplementary material.

Supplementary Material 1

## Data Availability

The site data used in this study is available online [https://github.com/mlee725/Spatiotemporal-patterns-in-air-pollution-and-sound-in-Dhaka-Bangladesh]. Additional data may be requested from corresponding authors for research purposes.
