# Supplementary material for: Spatiotemporal patterns in air pollution and sound in Dhaka, Bangladesh
Source: Sci Rep. 2025 Sep 25;15:32852. doi: 10.1038/s41598-025-12815-9 (PMC12464170; doi:10.1038/s41598-025-12815-9)
Supplement: Supplementary file 1 — Supplementary Material 1 [file 41598_2025_12815_MOESM1_ESM.pdf]

Supplementary Information for

Title: Spatiotemporal patterns in air pollution and sound in Dhaka, Bangladesh

Authors' names and affiliations

Martha Lee⊥ [1,2]

Anisur Rahman Bayazid⊥ [3]

Lauren Rosenthal [4]

Riaz Hossain Khan [3]

Raphael Arku [5]

Benjamin Barratt [2]

Zahidul Quayyum* [3]

Jill Baumgartner* [1,4,6]

[1] Department of Equity, Ethics, and Policy, McGill University, Montreal, Canada
[2] Environmental Research Group, MRC Centre for Environment and Health, Imperial College London, London, UK
[3] BRAC James P Grant School of Public Health, BRAC University, Dhaka, Bangladesh
[4] Department of Epidemiology, Biostatistics, and Occupational Health, McGill University, Montreal, Canada
[5] Department of Environmental Health Sciences, School of Public Health and Health Sciences, University of Massachusetts, Amherst, USA
[6] McGill Centre for Climate Change and Health, McGill University, Montreal, Canada

⊥Shared first authorship

***Corresponding authors:**

Jill Baumgartner: baumgartner@mcgill.ca
Zahidul Quayyum: zahidul.quayyum@bracu.ac.bd

Keywords: black carbon, fine particulate matter, inequalities, intermittency ratio, $PM_{2.5}$

Contents

Supplementary Table 1: Annual and seasonal levels of temporally-adjusted $PM_{2.5}$ and black carbon (BC) by site type and land use category - p.3

Supplementary Table 2: Differences in hourly $PM_{2.5}$ at fixed sites by season and day of the week: results from fitted linear regression models with cluster-robust standard errors - p.4

Supplementary Table 3: Sound metrics in Dhaka by site type and land use category - p.5

Supplementary Table 4: Noise Pollution Control Rules 2006 for Bangladesh - p.6

Supplementary Table 5: Differences in hourly sound at fixed sites by season and day of the week: results from fitted linear regression models with cluster-robust standard errors - p.7

Supplementary Table 6: Comparison of median intermittency ratio (IR) using +4 dBA cut-off to alternative cut-off values of +3 dBA and +5 dBA by site type and land use category - p.8

Supplementary Figure 1: Distributions of hourly average $PM_{2.5}$ by day of the week, season, and land use category - p.9

Supplementary Figure 2: Distributions of hourly average sound by day of the week and land use category - p.10

Supplementary Figure 3: Diurnal patterns of (A) average air pollution by season and (B) sound in Dhaka - p.11

Supplementary Figure 4: Correlation matrix of air pollution and sound measurements - p.12

Supplementary Note 1: Filter-based correction of sensor-based $PM_{2.5}$ measurements - p.13

Supplementary Note 2: Filter data processing - p.14

Supplementary Note 3: Performance comparison between sound monitors prior to field deployment - p.15

Supplementary Note 4: Seasonal trends in $PM_{2.5}$ and sound at fixed site locations - p.16

Supplementary Note 5: Seasonal adjustment of $PM_{2.5}$ and black carbon (BC) concentrations using a temporal adjustment factor (TAF) - p.18

Supplementary Note 6: Calculating the intermittency ratio - p.20

Supplementary Note 7: Comparison of intermittency ratio calculations using different event thresholds – p.21

Supplementary Table 1: Annual and seasonal levels of temporally-adjusted $PM_{2.5}$ and black carbon (BC) in Dhaka by site type and land use category. Data are expressed as the group medians of site averages for each category with standard error in parentheses and ranges in square brackets. Annual values are the average of the dry and wet season means at each site.

		$PM_{2.5}$ (µg/m^3)			BC (µg/m^3)	$BC/PM_{2.5}$ (%)
**Site type (no. of sites)**	**Season**	**All**	**Daytime 6:00-20:59**	**Night-time 21:00-5:59**	**All**	**All**
All sites (n=70)	Annual	117 (5.4) [80.5, 453]	112 (4.7) [81.8, 402]	128 (6.5) [78.3, 538]	8.07 (0.3) [2.94, 14.5]	6.35 (0.2) [3.13, 11.2]
	Dry	187 (5.4) [128, 479]	174 (4.3) [130, 415]	210 (6.7) [125, 585]	13.0 (0.5) [4.41, 23.7]	6.99 (0.3) [2.60, 15.0]
	Wet	47.8 (5.6) [27.4, 427]	45.8 (5.0) [29.0, 389]	48.0 (6.5) [24.5, 491]	2.61 (0.2) [1.18, 7.59]	5.33 (0.3) [1.78, 13.4]
Fixed sites (n=8)	Annual	119 (42.1) [109, 453]	112 (36.5) [99.8, 402]	127 (51.3) [117, 538]	9.36 (0.8) [7.15, 14.5]	7.31 (0.6) [3.13, 8.12]
	Dry	186 (37.0) [172, 479]	176 (30.6) [159, 415]	203 (47.6) [185, 585]	14.3 (1.2) [11.3, 21.5]	7.70 (0.6) [4.48, 9.56]
	Wet	48.6 (47.4) [42.5, 427]	48.6 (42.6) [41.2, 389]	48.6 (55.2) [44.8, 491]	3.23 (0.6) [2.29, 7.59]	6.28 (0.8) [1.78, 8.49]
Rotating sites (n=62)	Annual	116 (2.8) [80.5, 190]	110 (2.3) [81.8, 149]	128 (3.3) [78.3, 184]	7.84 (0.3) [2.94, 13.4]	6.22 (0.2) [3.14, 11.2]
	Dry	187 (3.9) [128, 318]	174 (2.9) [130, 249]	210 (4.4) [125, 294]	12.8 (0.6) [4.41, 23.7]	6.90 (0.3) [2.60, 15.0]
	Wet	47.7 (1.5) [27.4, 95.5]	45.2 (1.4) [29.0, 87.4]	48.0 (1.7) [24.5, 110]	2.57 (0.2) [1.18, 7.42]	5.21 (0.3) [2.29, 13.4]
Commercial / industrial (n=16)	Annual	125 (22.1) [100, 453]	117 (19.3) [97.7, 402]	135 (26.9) [105, 538]	9.44 (0.8) [4.75, 14.5]	6.64 (0.5) [3.13, 9.06]
	Dry	192 (19.5) [157, 479]	178 (16.1) [151, 415]	215 (25.5) [169, 585]	14.5 (1.4) [8.16, 23.7]	7.74 (0.6) [4.48, 11.6]
	Wet	52.9 (23.7) [27.4, 427]	52.2 (21.4) [29.0, 389]	54.4 (27.6) [24.5, 491]	3.06 (0.4) [1.34, 7.59]	4.93 (0.5) [1.78, 8.49]
Green, blue, open space (n=8)	Annual	113 (2.8) [99.7, 123]	106 (2.2) [97.0, 115]	124 (4.8) [104, 141]	8.56 (1.0) [2.94, 10.6]	7.14 (0.7) [3.14, 7.83]
	Dry	186 (5.9) [148, 200]	171 (4.6) [143, 186]	202 (10.4) [157, 236]	14.7 (1.8) [4.41, 19.1]	8.37 (1.0) [2.60, 9.96]
	Wet	45.4 (1.9) [33.1, 51.1]	43.9 (1.9) [34.3, 50.8]	46.6 (2.2) [31.1, 51.6]	2.11 (0.2) [1.47, 2.95]	4.85 (0.5) [3.24, 6.46]
Mixed-use zone (n=9)	Annual	113 (4.3) [100, 140]	108 (4.0) [95.6, 131]	124 (5.5) [103, 156]	7.49 (1.2) [4.00, 13.4]	6.46 (0.8) [3.60, 11.2]
	Dry	182 (6.9) [153, 213]	168 (5.8) [138, 194]	210 (9.8) [167, 251]	12.6 (1.7) [6.82, 23.0]	7.01 (1.1) [4.23, 15.0]
	Wet	47.8 (4.7) [33.2, 80.7]	45.2 (4.9) [33.4, 82.1]	47.9 (4.6) [32.7, 78.4]	3.14 (0.7) [1.18, 7.42]	6.57 (0.7) [2.97, 9.20]
Residential (n=19)	Annual	111 (3.8) [80.5, 140]	106 (3.3) [81.8, 138]	122 (4.8) [78.3, 159]	6.64 (0.4) [4.37, 10.9]	5.55 (0.4) [4.35, 9.77]
	Dry	183 (7.2) [128, 240]	169 (6.4) [130, 238]	202 (9.1) [125, 256]	11.1 (0.7) [7.54, 19.3]	6.42 (0.3) [4.28, 10.1]
	Wet	40.8 (2.1) [29.8, 67.3]	40.1 (2.0) [31.1, 63.9]	42.6 (2.4) [27.5, 72.4]	2.13 (0.2) [1.21, 5.56]	4.95 (0.6) [3.46, 13.4]
Transportation corridor (n=18)	Annual	123 (6.3) [100, 190]	115 (4.7) [95.1, 149]	134 (6.4) [110, 174]	8.54 (0.5) [4.86, 11.9]	6.51 (0.3) [4.34, 9.42]
	Dry	194 (9.8) [153, 318]	183 (6.4) [143, 249]	215 (9.1) [166, 294]	13.8 (0.9) [7.46, 21.2]	6.99 (0.5) [3.99, 10.7]
	Wet	49.7 (1.5) [42.6, 62.8]	48.8 (1.5) [44.0, 65.5]	52.8 (1.9) [39.2, 74.8]	3.40 (0.2) [1.34, 4.71]	6.15 (0.4) [3.06, 9.44]

Note: Site averages represent the mean of minute-level temporally-adjusted measurements.

Supplementary Table 2: Differences in hourly $PM_{2.5}$ at fixed sites by season and day of the week: results from fitted linear regression models with cluster-robust standard errors.

Variable	Season	Estimate	Confidence intervals
		Season	
Wet season (ref: Dry Season)		-126.3	-142.6, -110.0
		Weekday versus weekend	
Weekend days (ref: Weekdays)	Dry	4.6	-4.5, 13.6
	Wet	-0.7	-5.6, 4.3
		Day of the week	
Monday (ref: Sunday)	Dry	-14.4	-21.5, -7.3
	Wet	12.1	-8.2, 32.4
Tuesday	Dry	-27.9	-44.7, -11.2
	Wet	14.4	-10.9, 39.6
Wednesday	Dry	-18.6	-27.9, -9.4
	Wet	4.4	-1.4, 10.1
Thursday	Dry	-29.6	-37.1, -22.1
	Wet	-0.6	-3.0, 1.8
Friday	Dry	-9.8	-19.5, -0.1
	Wet	3.8	-3.5, 11.1
Saturday	Dry	-18.1	-33.6, -2.6
	Wet	7.1	-15.2, 29.3

Note: Analysis of 29611 hourly observations from 8 fixed sites (dry: 8670; wet: 10962) without temporal adjustment. Fridays and Saturdays were treated as weekend days while Sundays, Mondays, Tuesdays, Wednesdays, and Thursdays were treated as weekdays. We assessed potential differences in $PM_{2.5}$ between seasons and days of the week using a fitted linear regression model with cluster robust standard errors to account for correlation in site-specific repeated measurements.

Supplementary Table 3: Sound metrics in Dhaka by site type and land use category. Data are expressed as means (standard error) [ranges]. Wet and dry season data were combined for fixed sites.

**Land use category (no. of sites)**	**LA_{eq}[a] (dBA)**	**L_{Day}[b] (dBA)**	**L_{Night}[c] (dBA)**	**IR[d] (%)**	**IR_{Day}[e] (%)**	**IR_{Night}[f] (%)**
All sites (n=68)	64.7 (0.9) [50.5, 84.0]	65.9 (0.9) [50.5, 84.0]	61.5 (1.0) [48.5, 82.0]	49.9 (2.6) [9.11, 99.6]	47.0 (2.7) [6.99, 99.1]	51.5 (2.6) [13.6, 99.6]
Fixed sites (n=8)	63.6 (2.0) [58.0, 75.2]	64.8 (2.0) [60.4, 77.1]	59.9 (2.3) [52.8, 69.8]	39.7 (7.2) [19.8, 82.2]	36.2 (7.6) [17.3, 81.7]	39.3 (7.5) [16.6, 82.6]
Rotating sites (n=60)	64.9 (1.0) [50.5, 84.0]	66.0 (1.0) [50.5, 84.0]	61.7 (1.1) [48.5, 82.0]	51.3 (2.8) [9.11, 99.6]	48.4 (2.9) [6.99, 99.1]	53.2 (2.7) [13.6, 99.6]
Commercial / industrial (n=15)	65.0 (1.3) [57.2, 74.2]	66.3 (1.2) [60.2, 76.2]	61.4 (1.4) [52.8, 70.2]	52.9 (6.2) [21.0, 99.6]	50.0 (6.8) [13.7, 98.3]	55.5 (6.3) [16.6, 99.6]
Green, blue, open space (n=8)	55.2 (1.3) [50.5, 61.4]	56.6 (1.4) [50.5, 62.0]	52.5 (1.3) [48.5, 60.1]	63.6 (8.9) [19.8, 98.9]	61.2 (9.4) [17.7, 99.1]	58.2 (9.0) [20.7, 96.2]
Mixed-use zone (n=9)	71.2 (2.1) [60.9, 80.5]	72.1 (1.9) [62.8, 80.5]	68.0 (2.6) [54.9, 79.5]	35.1 (6.8) [9.11, 73.3]	32.3 (6.9) [6.99, 70.8]	38.1 (4.8) [13.6, 62.8]
Residential (n=19)	59.9 (1.0) [52.5, 68.9]	61.3 (1.0) [53.5, 70.9]	56.3 (0.9) [48.8, 63.9]	58.8 (3.1) [27.7, 78.6]	55.9 (3.4) [19.2, 82.0]	59.6 (4.4) [18.8, 85.4]
Transportation corridor (n=17)	70.8 (1.5) [60.9, 84.0]	71.7 (1.4) [62.2, 84.0]	68.0 (1.7) [55.2, 82.0]	38.7 (4.0) [17.6, 81.4]	35.3 (4.0) [9.97, 77.2]	42.9 (4.1) [20.6, 86.9]

[a]LA_{eq} is the median equivalent continuous sound level for the sampling period. [b]L_{day} is the median equivalent continuous daytime sound levels between 6:00 and 20:59. [c]L_{night} is the median equivalent continuous nighttime sound levels between 21:00 and 5:59. [d]IR_{24hr} is the median daily intermittency ratio. [e]IR_{Day} is the median daytime intermittency ratio between 6:00 and 20:59. [f]IR_{Night} is the median nighttime intermittency ratio between 21:00 and 5:59.

Supplementary Table 4: Noise Pollution Control Rules 2006 for Bangladesh

Land use type	**Noise limits (dBA)**	
	Day (6am – 8:59pm)	*Night 9pm – 5:59am)*
Silent zone	50	40
Residential	55	45
Mixed used	60	50
Commercial	70	60
Industrial	75	70

Note: Silent zones refer to areas within 100m meters of a hospital, school, or other so-designated institution (1)

Supplementary Table 5: Differences in hourly sound at fixed sites by season and day of the week: results from fitted linear regression models with cluster-robust standard errors.

Variable	Estimate	Confidence intervals
	Season	
Wet season (ref: Dry season)	-0.4	-1.3, 0.6
	Weekday versus weekend	
Weekend (ref: Weekday)	-0.2	-0.7, 0.3
	Day of the week	
Monday (ref: Sunday)	0.6	0.2, 1.1
Tuesday	0.7	0.1, 1.3
Wednesday	0.8	0.5, 1.2
Thursday	0.4	-0.1, 0.8
Friday	0.6	0.1, 1.1
Saturday	0.0	-0.6, 0.6

Note: Analysis of 15378 hourly observations from 8 fixed sites (dry: 4118; wet: 11260). Fridays and Saturdays were treated as weekend days while Sundays, Mondays, Tuesdays, Wednesdays, and Thursdays were treated as weekdays. We assessed potential differences in LA_{eq} between seasons and days of the week using a fitted linear regression model with cluster robust standard errors to account for correlation in site-specific repeated measurements.

Supplementary Table 6: Comparison of median intermittency ratio (IR) using +4 dBA cut-off to alternative cut-off values of +3 dBA and +5 dBA by site type and land use category. Data are expressed as medians (interquartile ranges)[a]. Wet and dry season data were combined for fixed sites.

	+4 dBA (main analysis)			+3 dBA			+5 dBA		
**Land use category (no. of sites)**	**IR[a] (%)**	**IR_{Day}[b] (%)**	**IR_{Night}[c] (%)**	**IR[a] (%)**	**IR_{Day}[b] (%)**	**IR_{Night}[c] (%)**	**IR[a] (%)**	**IR_{Day}[b] (%)**	**IR_{Night}[c] (%)**
All sites (n=68)	49.1 (29.9)	47.9 (32.1)	48.4 (28.5)	53.4 (26.5)	52.1 (28.8)	54.8 (26)	43.7 (30.9)	41.4 (32.9)	43.2 (29.2)
Fixed sites (n=8)	35.5 (20.7)	36.3 (21.7)	35.1 (20.9)	42.5 (22.5)	40.1 (18.2)	41.8 (18.1)	31.9 (21.7)	30.8 (24.0)	29.9 (20.8)
Rotating sites (n=60)	50.1 (30.2)	48.6 (31.3)	50.9 (25.7)	57.4 (28.2)	53.3 (28.8)	56.6 (23.3)	46.7 (31.2)	43.9 (31.1)	44.1 (30.8)
Commercial / industrial (n=15)	49.2 (25.9)	48.5 (27.2)	52.2 (16.4)	58.9 (21.2)	55.6 (26.1)	63.1 (16.2)	43.0 (27.1)	40.0 (29.3)	46.1 (24.5)
Green, blue, open space (n=8)	68.2 (32.6)	67.4 (33.0)	52.8 (34.9)	71.5 (29.8)	71.7 (32.6)	58.1 (32.7)	64.2 (32.4)	62.4 (31.9)	49.2 (36.5)
Mixed-use zone (n=9)	35.5 (31.3)	32.8 (33.8)	36.7 (14.9)	42.2 (29.3)	38.8 (30.9)	48.9 (12.3)	29.1 (31.5)	29.4 (32.8)	31.9 (18.8)
Residential (n=19)	61.3 (16.8)	55.8 (15.4)	64.2 (23.2)	65.0 (14.1)	59.9 (15.8)	66.5 (21.8)	58.0 (20.0)	53.7 (14.6)	59.6 (28.4)
Transportation corridor (n=17)	35.3 (17.2)	32.2 (17.9)	41.5 (10.8)	44.0 (17.5)	37.9 (13.6)	46.0 (11.3)	29.8 (21.2)	27.8 (25.9)	35.6 (14.2)

[a]IR is the median intermittency ratio. [b]IR_{Day} is the median daytime intermittency ratio between 6:00 and 20:59. [c]IR_{Night} is the median nighttime intermittency ratio between 21:00 and 5:59.

Supplementary Figure 1: Distributions of hourly average $PM_{2.5}$ by day of the week, season, and land use category. Data is expressed as hourly means to visually examine weekly temporal trends across season and land use category.

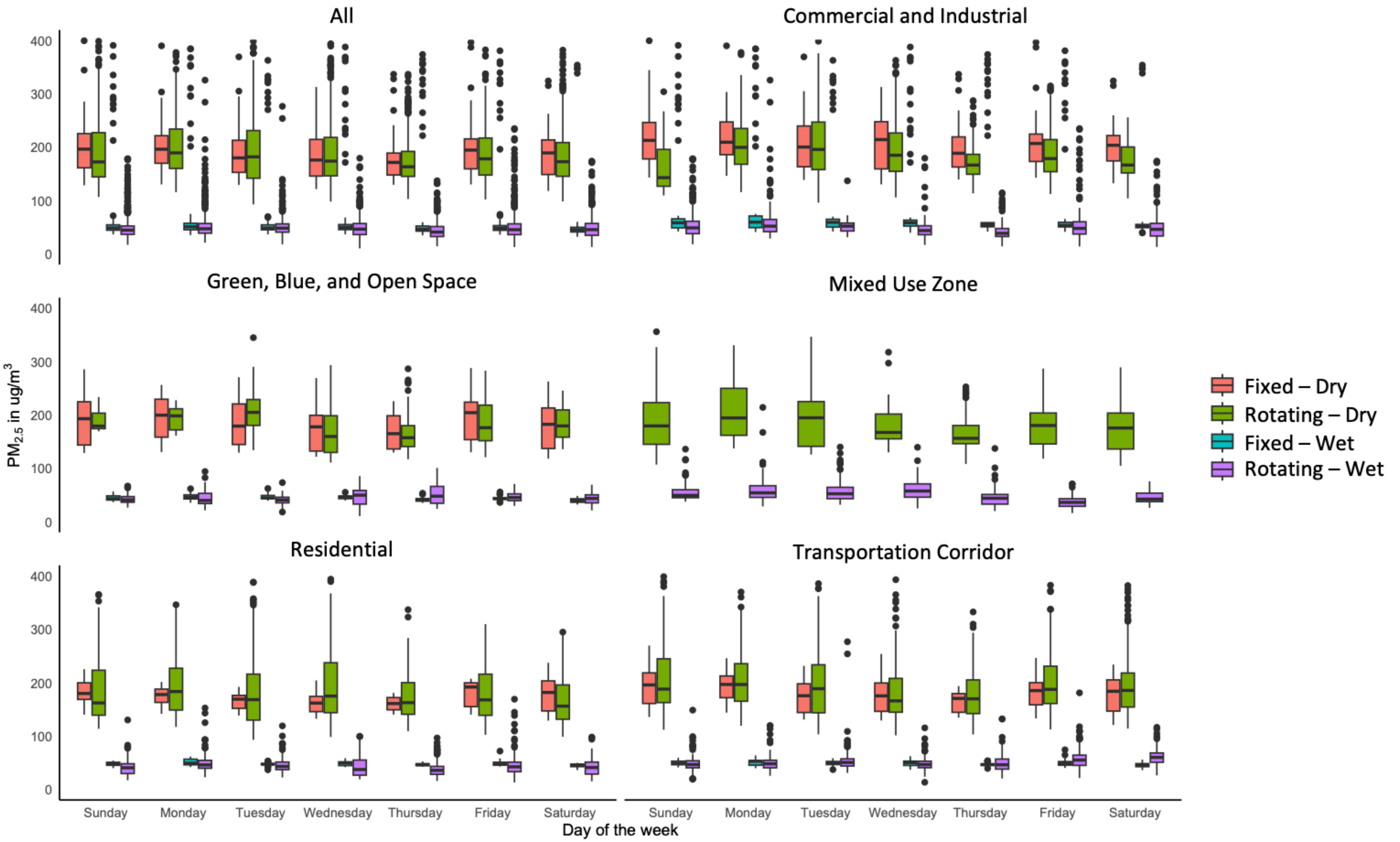

Note: For visual ease, the y-scale was limited to 400 µg/m³ meaning 228 hourly site averages, ranging from 400.2 to 986.6 µg/m³, were excluded from this figure. The majority (n = 206) of these hourly averages were from Shyampur, an industrial site, while five of the other excluded hourly averages from four other commercial and industrial sites. Six transportation corridor sites accounted for 12 of the outliers and one residential site accounted for the remaining five.

Supplementary Figure 2: Distributions of hourly average sound by day of the week and land use category. Data is expressed as hourly medians to visually examine weekly temporal trends across land use category.

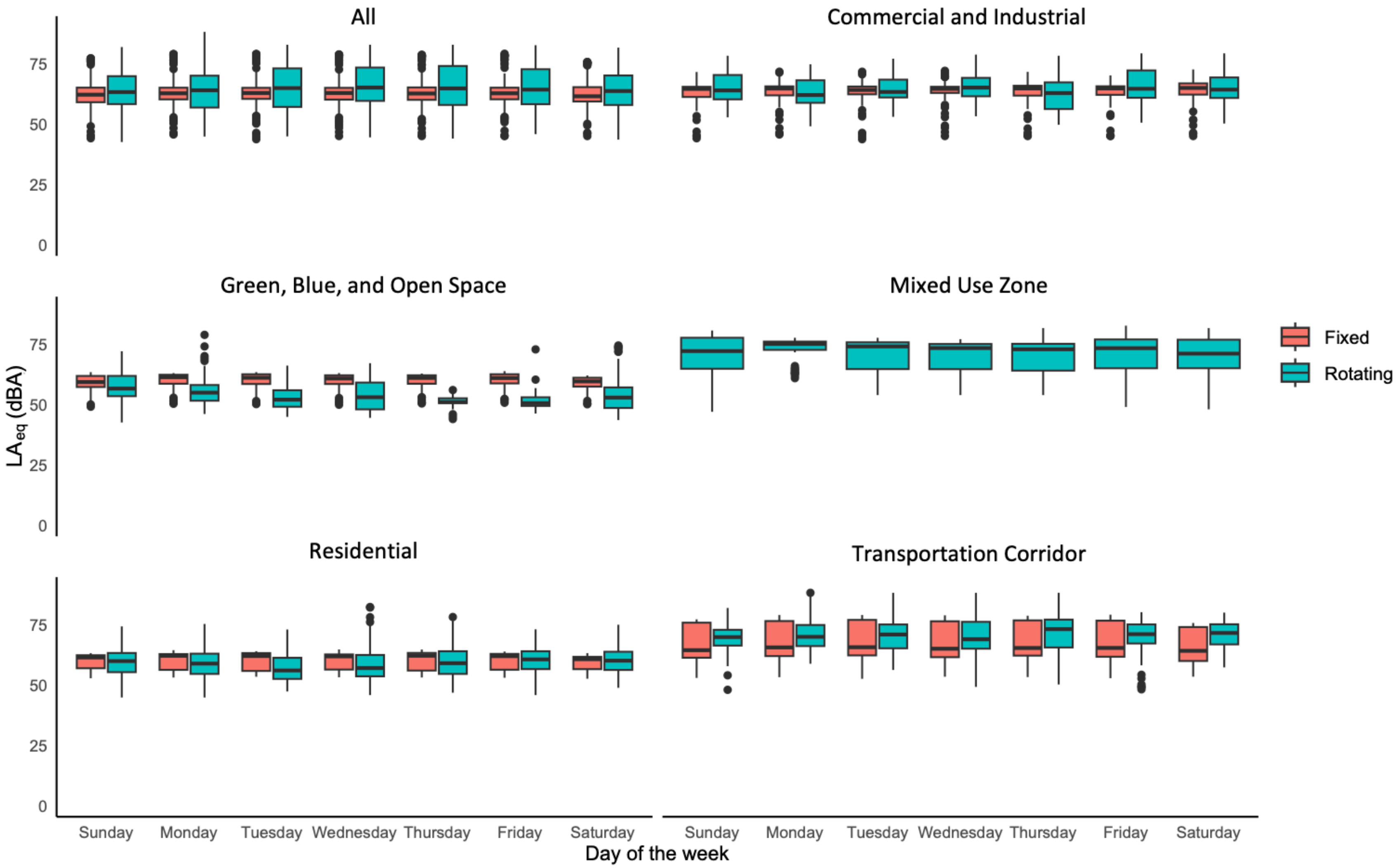

Supplementary Figure 3: Diurnal patterns of (A) average air pollution by season and (B) sound in Dhaka. Air pollution data shows the filter- and temporally-adjusted mean $PM_{2.5}$ ($\mu g/m^3$) by season and land use category. Sound data shows the site averaged median LA_{eq} throughout the day by land use category. Minute-level data smoothed using 15-minute rolling averages.

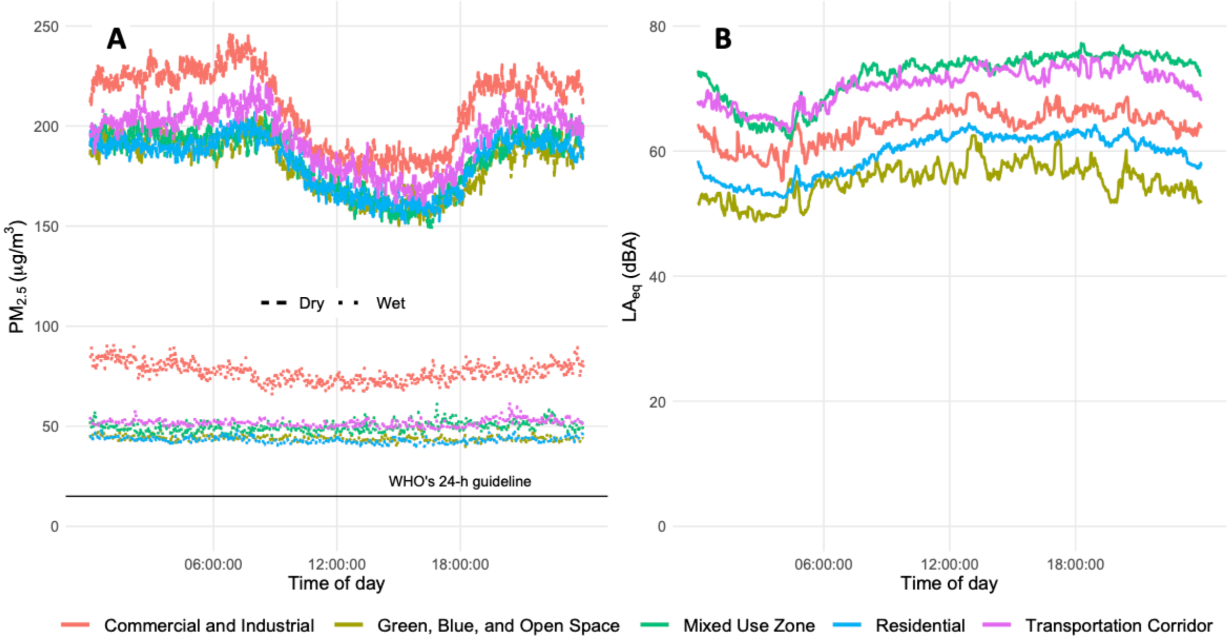

Supplementary Figure 4: Correlation matrix of air pollution and sound measurements

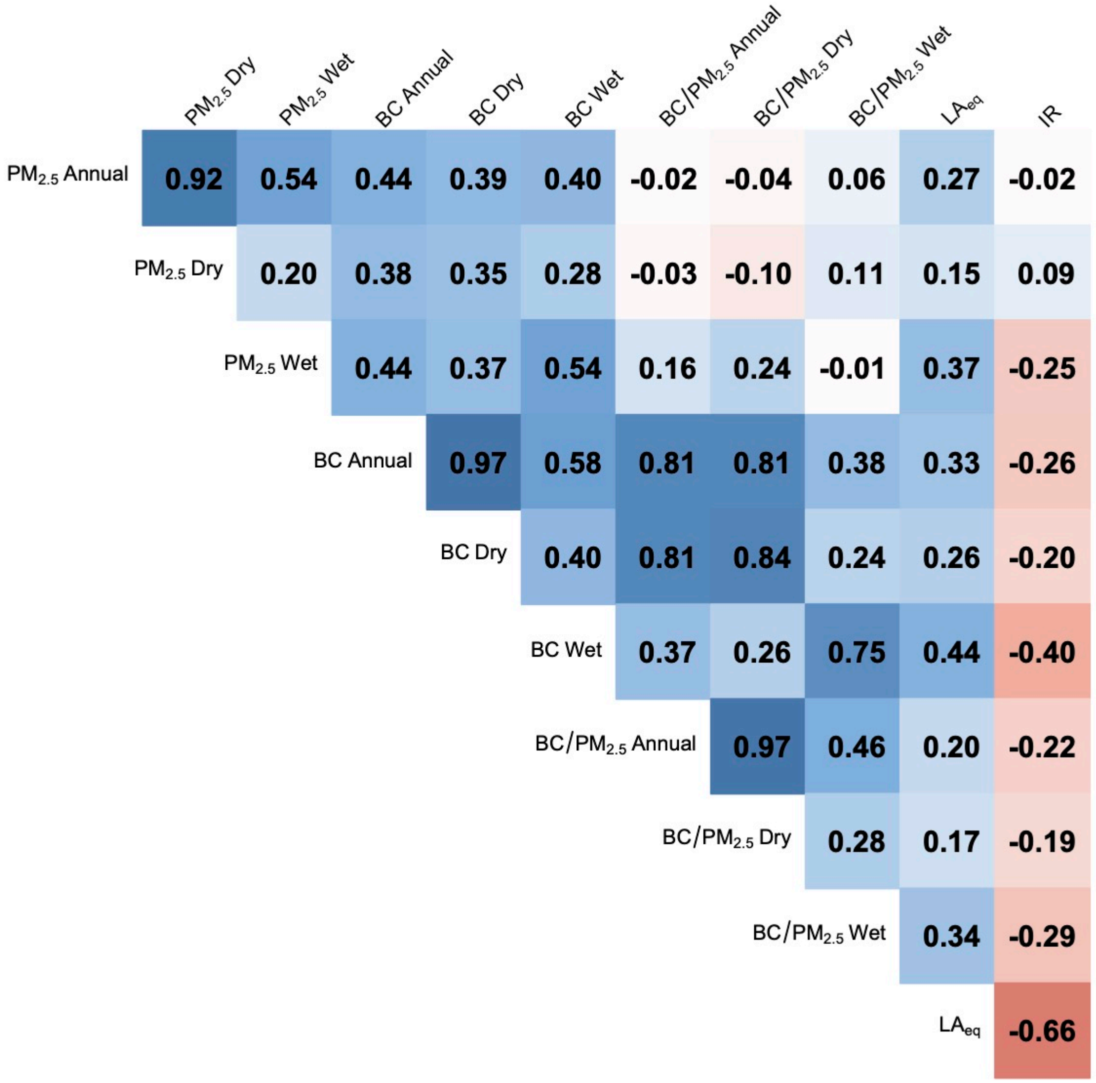

Note: Correlations were computed based on one median value per site. Annual $PM_{2.5}$, BC, and BC/$PM_{2.5}$ refers to the averaged seasonal concentration for each site, for each pollutant, respectively. Dry and wet $PM_{2.5}$, BC, and BC/$PM_{2.5}$ refers average concentration at each site in the dry and wet seasons for each pollutant, respectively. LA_{eq} and IR refer to the median values for each site based on both seasons' data combined. All $PM_{2.5}$ and BC data is temporally adjusted.

Supplementary Note 1: Filter-based correction of sensor-based $PM_{2.5}$ measurements

We adjusted sensor-based $PM_{2.5}$ measurements to match filter-based $PM_{2.5}$ mass (the reference concentration) using data from co-located sensor and gravimetric measurements. For each season, we applied a linear regression model to assess the relationship between these two measurements, ensuring that the averaging period for sensor data matched the runtime of the corresponding gravimetric measurement. Separate seasonal adjustments were created for Shyampur. Outliers were identified through visual inspection and excluded (n = 7 in the dry season and 6 in the wet season). We also removed samples where the sensor operated for <90% of the UPAS runtime (n = 15 in the dry season and 2 in the wet season). The resulting equations, shown in red, text in the figures below, were used to correct the sensor-based data.

Relationships between filter-based $PM_{2.5}$ and sensor-based $PM_{2.5}$ measurements using co-located monitors in the dry (left) and wet (right) seasons.

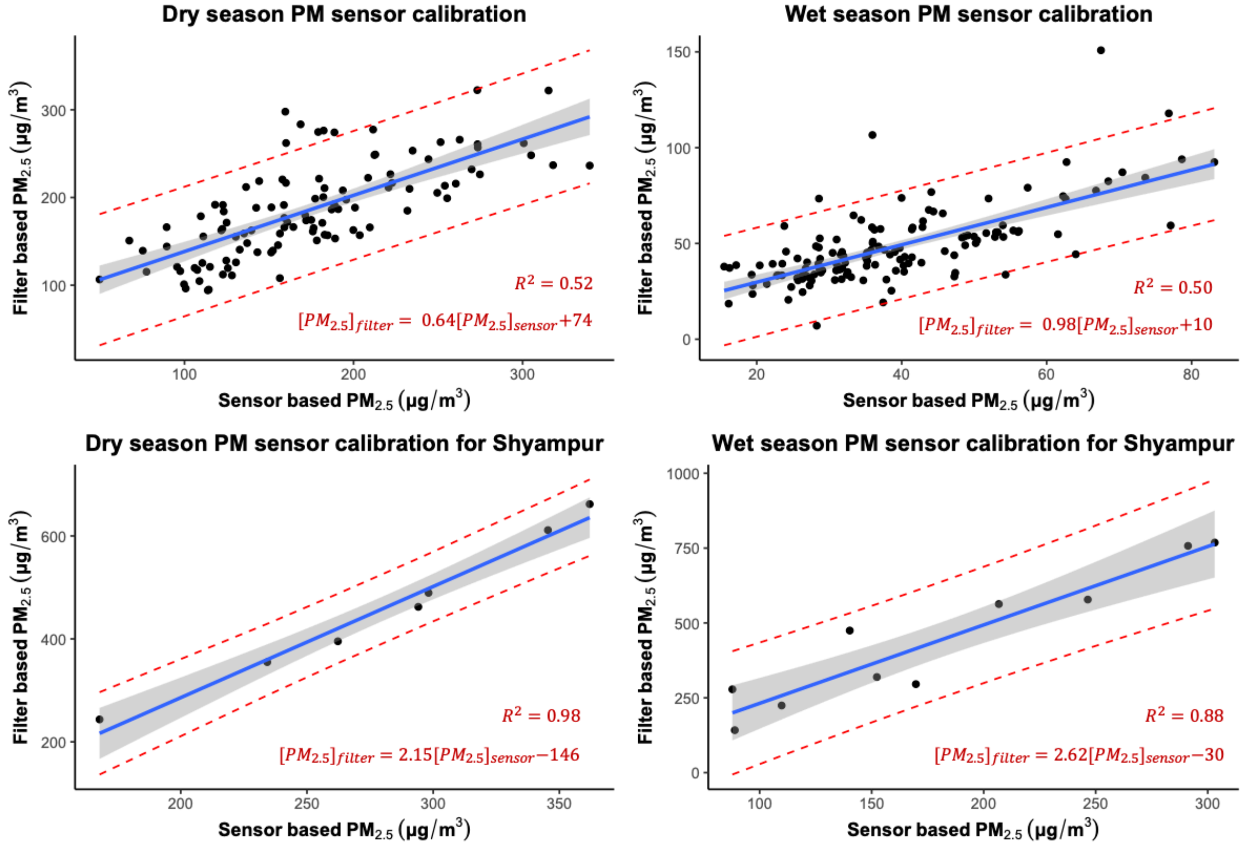

Supplementary Note 2: Filter data processing

The raw filter-based fine particulate matter ($PM_{2.5}$) data were blank-corrected and converted to concentrations (µg/m^3) as follows:

$$Concentration\ (\mu g/m^3) = \frac{raw\ data\ (\mu g) - median\ blank\ reading\ (\mu g)}{volume\ of\ air\ drawn\ through\ monitor\ (m^3)}$$

Post blank correction, the optical attenuation BC data were converted to total filter mass using the classical Magee-recommended mass absorption cross-section of 16.6 m^2/g for an 880 nm channel. The conversion was done as follows (equation recreated from Presler-Jur et al. 2017) (2):

$$BC_{filter\ mass}\ (\mu g) = \frac{b_{att}(Mm^{-1})}{\sigma_{att}[\lambda]\left(\frac{m^2}{g}\right)} * A_{sampled}(cm^2)$$

Where BC is black carbon (µg), b_{att} is the optical attenuation at 880 nm (Mm^{-1}), σ_{att} is the mass absorption cross-section (m^2/g) at λ = 880 nm, and $A_{sampled}$ is the sampled surface area of the filters. The area of the filter was 10.8 cm^2 but the sampled surface area was 8.6 cm^2 as the UPAS O-ring covered a proportion of the filter. Like $PM_{2.5}$, BC concentrations were calculated by dividing the $BC_{filter\ mass}$ by the volume (m^3) of air drawn through the monitor.

Supplementary Note 3: Performance comparison between sound monitors prior to field deployment

We co-located the sound monitors prior to data collection to evaluate comparability across the three monitor types and identify any poor-performing monitors. Comparison of real-time (minute-level) sound levels (dBA) for NS110 and NSRT_mk4 monitors (both only used in the wet season) and a NSRT_mk3 monitor during a 20 h test period revealed that measurements were within +/- 1.5 dBA of the median, except for one monitor (C) which was subsequently excluded from data collection.

Comparison of sound levels (dBA) between NS110 and NSRT_mk4 monitors (A through J) and a NSRT_mk3 monitor.

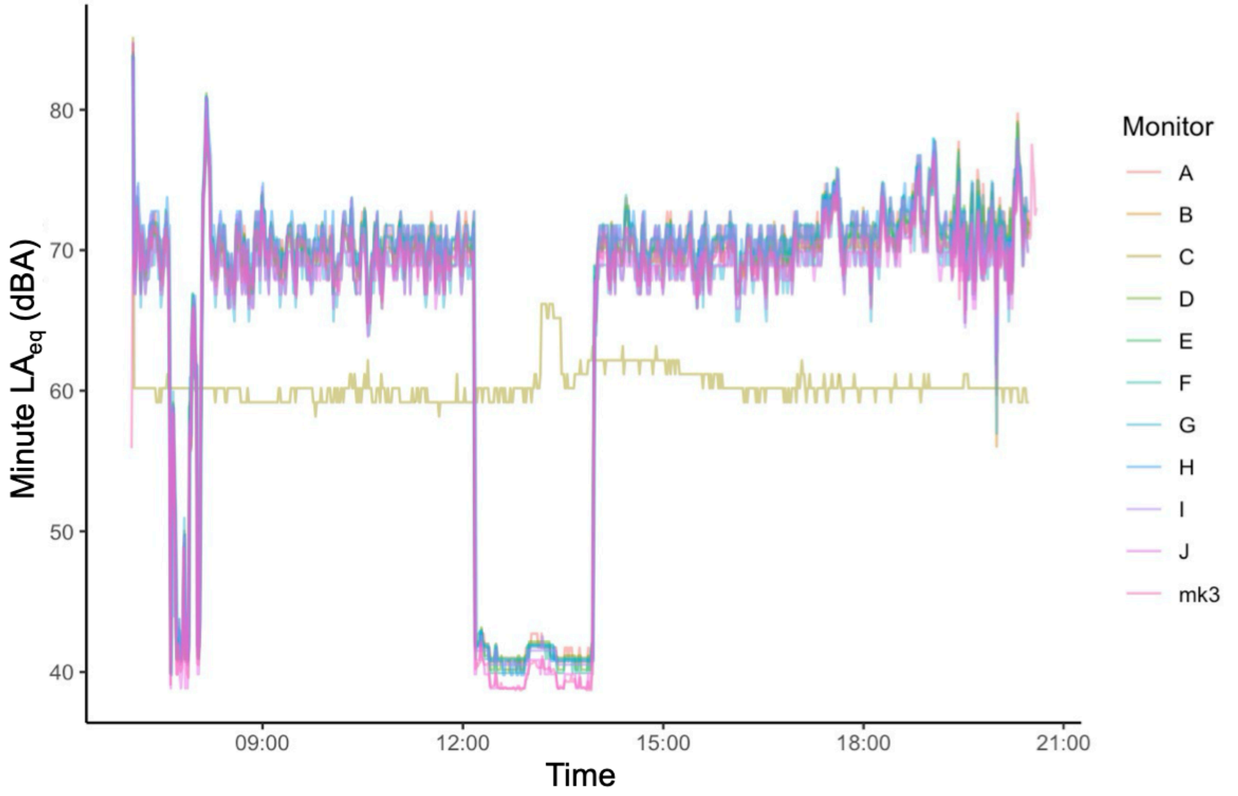

Supplementary Note 4: Seasonal trends in $PM_{2.5}$ and sound at fixed site locations

We observed a strong temporal pattern in $PM_{2.5}$ in the dry season where concentrations steadily decreased over time. The wet season concentrations slightly increased and then decreased over time, though much less pronounced than differences observed in the dry season.

Trends of weekly averaged filter-adjusted sensor-based $PM_{2.5}$ data from the fixed site locations in the dry season and wet season.

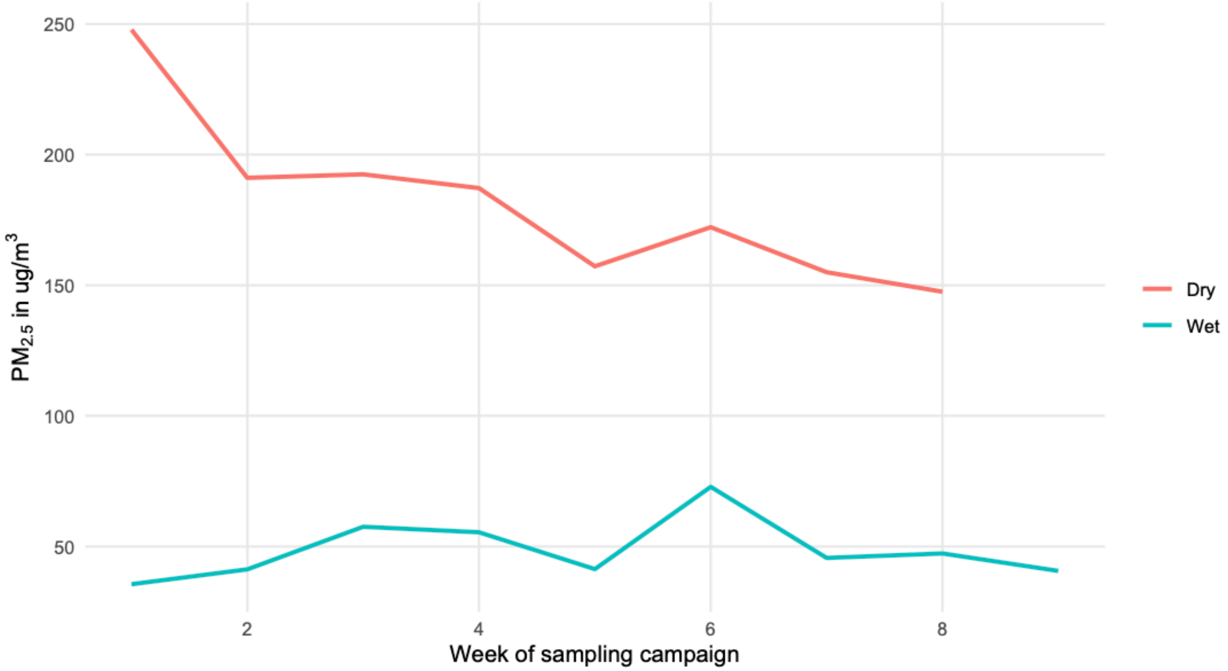

Note: The Shyampur fixed site was excluded because air pollution trends did not follow the same seasonal trends other sites.

In comparison sound exhibited little seasonal with similar distributions of measurements from fixed sites in both seasons.

Distribution of hourly sound measurements in the dry (January to March) and wet (June to August) seasons at fixed sites

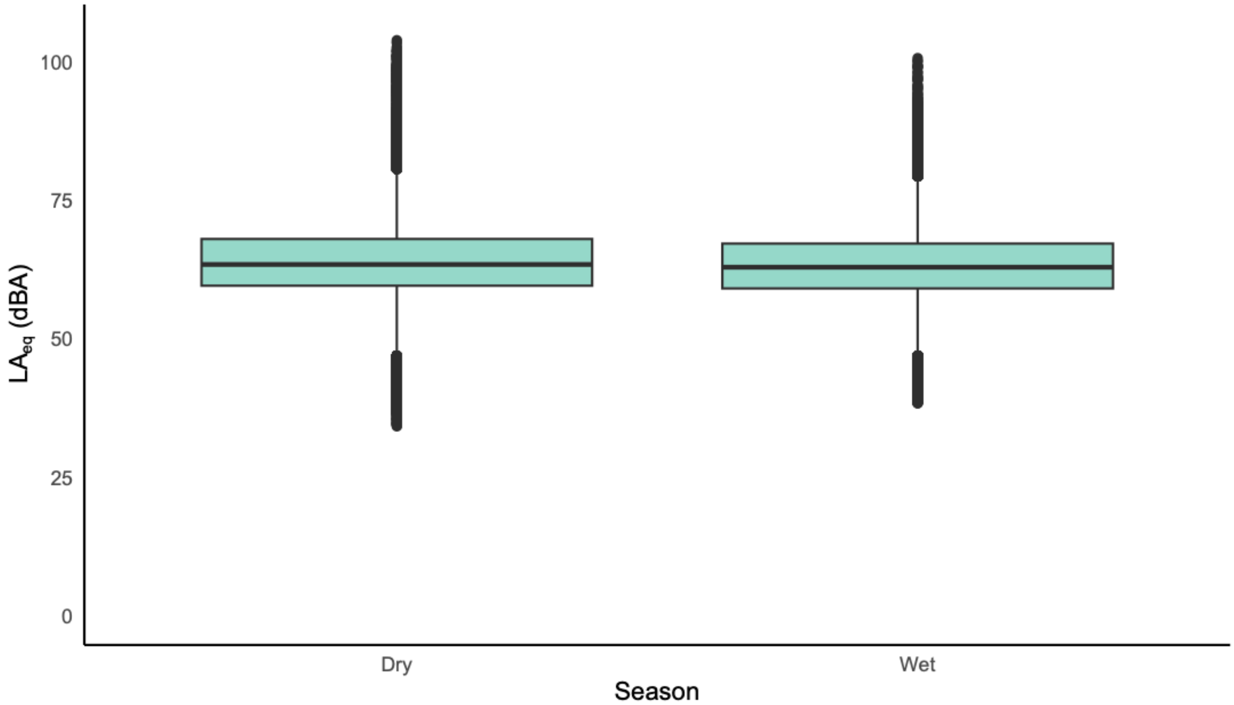

Note: A boxplot was used as the limited number of sound meters available in the dry season meant fluctuations in weekly averaged were a function of what sites were being sampled rather than a temporal trend.

Supplementary Note 5: Seasonal adjustment of $PM_{2.5}$ and black carbon (BC) concentrations using a temporal adjustment factor (TAF)

Seasonal adjustment of the $PM_{2.5}$ data was done using a temporal adjustment factor (TAF) also described in Alli et al. 2021 (3). Briefly, for each week of data collection, we calculated the ratio of the mean $PM_{2.5}$ concentration across the fixed sites and the US embassy rooftop site during that week to the mean $PM_{2.5}$ concentration across these sites for the entire sampling season. We excluded the industrial site (Shyampur) which showed a different temporal trend and much higher concentrations of air pollution than other fixed sites. The same method was used for BC, excluding the US embassy site as this monitor does not provide BC data. This ratio was used for adjustment based on the week when sampling occurred, using the following equation:

$$(\boldsymbol{C_i})_{\boldsymbol{j}}^{\boldsymbol{adjusted}} = \frac{(\boldsymbol{C_i})_{\boldsymbol{j}}}{(\boldsymbol{C^{week}})_{\boldsymbol{j}} / \boldsymbol{C^{season}}}$$

Where C_i is the concentration measured at a given site during the *j*th week of the year, $(C^{week})_j$ is the mean concentration measured at the fixed and embassy sites during the *j*th week of the year, and (C^{season}) is the mean concentration measured at the fixed and embassy sites over the entire season.

Distributions of fixed site $PM_{2.5}$ measurements. The boxplots represent the distribution of (A) differences in $PM_{2.5}$ concentrations (μg/m³), corrected using gravimetric and TAF methods, measured during the first 72 hours of each 5-day monitoring period at fixed sites (n=9-12 per fixed site per season) with the site-specific seasonal means, i.e., the average filter-corrected $PM_{2.5}$ concentrations (μg/m³) over the full campaign period at each fixed site, and (B) the same 72 hours $PM_{2.5}$ concentrations (μg/m³) relative to the site-specific seasonal means (the red dots).

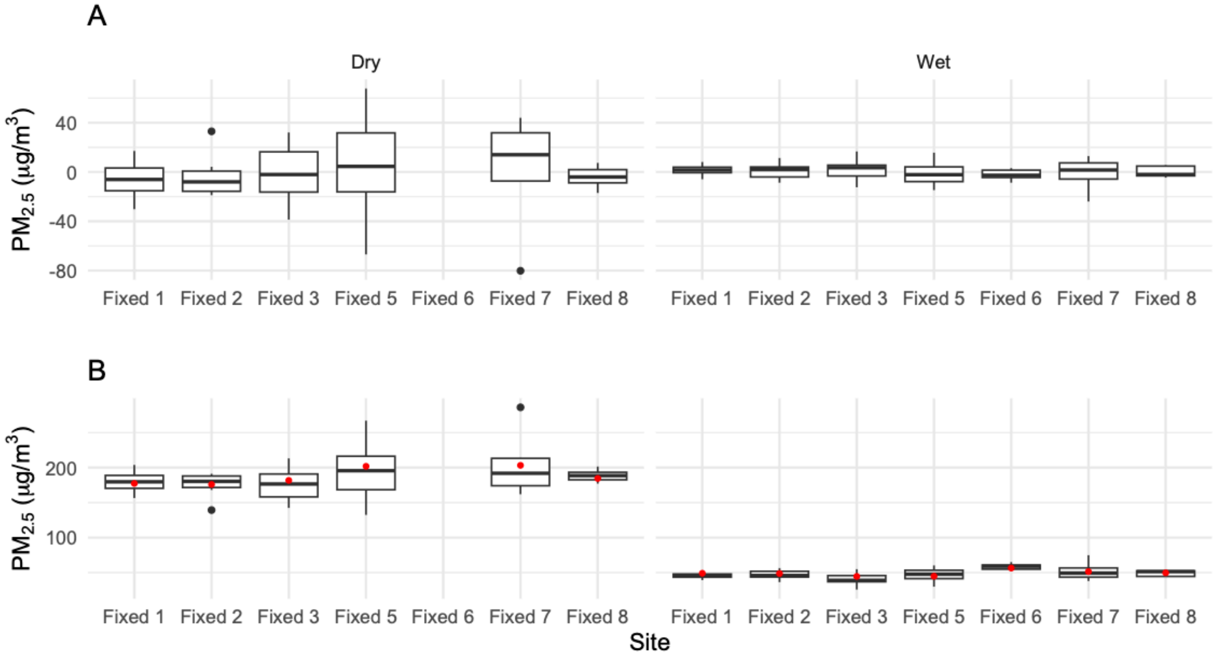

Note: Fixed site 6 (dry season) was excluded as this site was deployed during the 3rd week of that sampling campaign. Shyampur (fixed site 4) was also removed as this site was excluded from creation of the TAF due to having different temporal trend than other fixed sites.

We found the mean RMSE across fixed sites by season when comparing first 72 hours TAF-adjusted measurements with the site-specific seasonal means was 22.3 μg/m³ (dry) and 6.4 μg/m³ (wet) with a range of 8.7 to 37.1 μg/m³ and 3.8 to 10.7 μg/m³, respectively.

Supplementary Note 6: Calculating the intermittency ratio

The intermittency ratio (IR) was calculated using the equations presented in Wunderli et al. (2016) (4):

$$IR = \frac{10^{0.1\,L_{eq,T,events}}}{10^{0.1\,L_{eq,T,tot}}} \cdot 100$$

Where:

$$L_{eq,T,tot} = 10\,log_{10}\left(\frac{1}{T}\int_0^T 10^{0.1L(t)}dt\right)$$

is the overall sound pressure level (i.e. background sound level) at a sampling site calculated using the continuous sound pressure level measurements (L(t)) collected at sites over a given time period (T) and

$$L_{eq,T,events} = 10\,log_{10}\left(\frac{1}{T}\int_0^T H(L(t) - (L_{eq,T,tot} + C))10^{0.1L(t)}dt\right)$$

is the event-based sound pressure level, which captures sound events during a given time period based on measurements that exceed the background sound level by a selected threshold(C) where the Heaviside step function (H) is equal to 1.

Supplementary Note 7: Comparison of intermittency ratio calculations using different event thresholds

In order to characterize a noise event for the intermittency ratio (IR) calculation, the equivalent continuous sound level must surpass a background equivalent sound level by a given threshold. The standard threshold value is 3 dBA (Wunderli et al., 2016) (4), but we elected for a higher threshold of 4 dBA given Dhaka's particularly high levels of background sound, where a higher level of sound was required in order to qualify as a disruptive "event". The mean absolute difference in IR values of using 3 versus 5 dBA thresholds were 5.1 and 4.8% respectively when comparing the median site values (n = 68). We observed a high Pearson correlation (r = 0.99) between our threshold of 4 dBA in the main analysis and the 3 and 5 dBA threshold values in sensitivity analyses.

Works Cited

1. Government of the People's Republic of Bangladesh. Noise Pollution Control Rules, 2006. In: Ministry of Environment and Forest, editor. 2006.
2. Presler-Jur P, Doraiswamy P, Hammond O, Rice J. An evaluation of mass absorption cross-section for optical carbon analysis on Teflon filter media. Journal of the Air & Waste Management Association. 2017;67(11):1213-28.
3. Alli AS, Clark SN, Hughes A, Nimo J, Bedford-Moses J, Baah S, et al. Spatial-temporal patterns of ambient fine particulate matter (PM2.5) and black carbon (BC) pollution in Accra. Environmental Research Letters. 2021;16(7).
4. Wunderli JM, Pieren R, Habermacher M, Vienneau D, Cajochen C, Probst-Hensch N, et al. Intermittency ratio: A metric reflecting short-term temporal variations of transportation noise exposure. Journal of exposure science & environmental epidemiology. 2016;26(6):575-85.
